# Supplementary material for: A survey on the attitudes of parents with young children on in-home monitoring technologies and study designs for infant research
Source: PLoS One. 2021 Feb 5;16(2):e0245793. doi: 10.1371/journal.pone.0245793 (PMC7864397; doi:10.1371/journal.pone.0245793)
Supplement: S1 File — (PDF) [file pone.0245793.s001.pdf]

S1 File

A survey on the attitudes of parents with young children on in-home monitoring technologies and study designs for infant research.

Supplementary Information S1

Smart Suits Study

Information and consent

Before you decide to take part in this survey, it is important for you to understand why the research is being done and what it will involve. Please take the time to read the following information carefully and discuss it with others if you wish. A member of the research team can be contacted via email if there is anything that is not clear or if you would like more information. Take time to decide whether or not you wish to take part.

We are currently in the process of designing a new study to investigate how infants behave at home. To help us do this, we want to know what parents would find acceptable and practical. This survey asks for your thoughts on some of our proposed measures. Please note that by completing this survey you are not obliged to participate in our new proposed study. All questions are hypothetical and are designed to help us understand what parents of young children think.

Result from this survey will be used to guide the design of our new study. We might write up the results for publication to help other researchers understand what measures parents might be comfortable with.

Answers will be analysed as a group, and all data will be totally anonymous, without any means of identifying the individuals involved. At the end of the study you have the option to not submit your answers and withdraw from participation. However, as submissions are anonymous you will be unable to withdraw your answers once they have been submitted.

The project has received ethical approval from the Department of Psychological Sciences Research Ethics Committee of Birkbeck University of London
If you wish to seek more information in regards to the survey please contact Laurel Fish via email (ublfis002@mail.bbk.ac.uk).

* 1. I have read the details of the survey and willingly consent to take part.
I understand that I may ask further questions at any time via email.
I understand that my answers will remain anonymous and that all the information given will be used to help researchers design studies with parents and infants. If results are written up for publication, no individuals will be identified.
I understand that I may withdraw my consent for the survey at any time without giving any reason and to decline to answer particular questions. Furthermore I understand that I will be unable to withdraw my answers for the survey after I have submitted them as the answers are anonymised.
I understand that all information given will be kept confidential. All data will be identified by a code, with personal details kept in a secure computer with access only by the immediate researchers.
I understand my answers will be used to help guide the design of a new study.

- ◯ I agree
- ◯ I do not agree

Smart Suits Study

About the proposed study

Due to the development of new technologies, we are able to investigate the behaviour of infants in the home. With such technologies we are able to discretely and precisely measure factors such as heart rate, movement, vocalisations and sleep cycles for extended periods of time. This could help us understand the development of babies who are developing typically, and those who might be more likely to develop difficulties. To help us plan our new study, we want to know your thoughts on studies with young children. Our proposed study will be designed to collect data on babies and toddlers aged 3-24 months. Therefore, if your youngest child is now older please consider the following questions for when your child was between 3-24 months.
Please note that by answering the below questions in no way means that you are obliged to participate in the proposed study. The purpose of this survey is to find out the thoughts of parents with a young child to facilitate the designing process of our new study.

Smart Suits Study

Demographics

First, we would like to know a little about your youngest child and the rest of your

family.

* 2. What is your relationship to your child?

- [ ] Mother
- [ ] Father
- [ ] Grandparent
- [ ] Prefer not to answer
- [ ] Other (please specify)

* 3. How old is your **youngest child (in months)**?

* 4. What is your **youngest child's** sex?

- ( ) Female
- ( ) Male
- ( ) Non-binary
- ( ) Prefer not to answer

* 5. One of the goals of our study would be to learn more about infants or children with developmental disorders. So, we want to know how families who have experience with developmental conditions might feel about our proposed study.

Our planned study wouldn't help us diagnose particular developmental conditions in children. However, we hope it might help us learn more about the difficulties experienced by children with developmental conditions.

Therefore we would like to know if your**youngest child** has any of the following conditions.

- [ ] Autism Spectrum Disorder
- [ ] ADHD
- [ ] I don't know
- [ ] No
- [ ] Other medical condition
- [ ] Prefer not to answer

* 6. How would you describe your **youngest child's** ethnicity?

- White
- Asian
- Black
- Mixed race
- Prefer not to answer
- Other (please specify)

* 7. Does your **youngest child's** immediate family have any of the following disorders.

	Mother	Father	Siblings	1/2 Siblings	First Cousins	Aunts/Uncle	Grand-parents
Autism Spectrum Disorder	☐	☐	☐	☐	☐	☐	☐
Genetic Syndrome	☐	☐	☐	☐	☐	☐	☐
Attention Deficit Disorder (ADHD)	☐	☐	☐	☐	☐	☐	☐
Anxiety Disorder	☐	☐	☐	☐	☐	☐	☐
Depression	☐	☐	☐	☐	☐	☐	☐
Other	☐	☐	☐	☐	☐	☐	☐
None	☐	☐	☐	☐	☐	☐	☐
Prefer not to answer	☐	☐	☐	☐	☐	☐	☐

Smart Suits Study

Demographics continued

Here, we would like to know a bit more about who cares for your child to figure out whether our study would be appropriate for families with a wide range of circumstances.

* 8. Please check the range that best describes your annual household income

- < £20 000
- £20 000 - £29 999
- £30 000 - £39 999
- £40 000 - £59 999
- £60 000 - £79 999
- £80 000 - £99 999
- £100 000 - £149 999
- > £149 999
- Do not wish to answer

* 9. Who is the primary care giver for your youngest child on each day of the week?

	Monday	Tuesday	Wednesday	Thursday	Friday	Saturday	Sunday
Mother	☐	☐	☐	☐	☐	☐	☐
Father	☐	☐	☐	☐	☐	☐	☐
Grandparent	☐	☐	☐	☐	☐	☐	☐
Nanny	☐	☐	☐	☐	☐	☐	☐
Nursery/Day care	☐	☐	☐	☐	☐	☐	☐
Other	☐	☐	☐	☐	☐	☐	☐
Prefer not to answer	☐	☐	☐	☐	☐	☐	☐

* 10. What is the highest level of education gained by your youngest child's mother?

- Primary (e.g. Primary school education)
- Secondary (e.g. GCSE, A-levels or equivalent)
- Tertiary (e.g. Undergraduate/Postgraduate degree)
- Don't know
- Prefer not to answer

* 11. What is the highest level of education gained by your youngest child's father?

- ◯ Primary (e.g. Primary school education)
- ◯ Secondary (e.g. GCSE, A-levels or equivalent)
- ◯ Tertiary (e.g. Undergraduate/Postgraduate degree)
- ◯ Don't know
- ◯ Prefer not to answer

Smart Suits Study

Smart Suits

Some of the measures in our proposed study will require the infant to wear ‘smart suits’. These are specially designed baby rompers that are comfortable for your child to wear. They are made from special fabric with the technology to measure arousal levels. During the proposed study we will provide all the vests that would be needed for the whole study. Smart suits are machine washable.
If your youngest child is now older please consider the following questions for when your child was between 3-24 months.

Here is an example of how the smart suit technology may look.

Image Removed

* 12. How likely would you be to allow your child to wear one of our smart vests?

- ◯ Extremely likely
- ◯ Very likely
- ◯ Moderately likely
- ◯ Slightly likely
- ◯ Not at all likely
- ◯ Prefer not to answer

* 13. How long will you be willing to participate in the smart suits part of the proposed study?

- ◯ Not at all
- ◯ A one off (e.g. for an afternoon or 1 day)
- ◯ Once every now and then (e.g. once a week or once a month)
- ◯ Continuously for a weekend
- ◯ Continuously for a week
- ◯ Continuously for a month
- ◯ Prefer not to answer

* 14. How practical would you find dressing your child in the above described 'smart suits' once (e.g for an afternoon or day)?

- ☐ Practical
- ☐ Somewhat practical
- ☐ Somewhat impractical
- ☐ Not at all practical
- ☐ Prefer not to answer
- ☐ Comments

15. How practical would you find dressing your child in the above described 'smart suits' over a long time (e.g. a week/month)?

☐ Practical

☐ Somewhat practical

☐ Somewhat impractical

☐ Not at all practical

☐ Prefer not to answer

☐ Comments

* 16. Would you allow your child to wear vests other families have used?

○ Yes

○ No

○ Prefer not to answer

17. Do you have any comments about the proposed smart suits?

Smart Suits Study

Heart rate and sweat response stickers

Alternatively to the smart suits, during the proposed study we could provide you with stickers that are to be placed on the infants back directly on their skin. These stickers are attached to wires connected to a small device to be carried in the infants pocket. The stickers would measure heart rate and sweat levels to assess arousal levels.
If your youngest child is now older please consider the following questions for when your child was between 3-24 months.

Here is an image of the sticker technology.

Image Removed

* 18. How likely would you be to allow your child to wear the above described stickers?

- ◯ Extremely likely
- ◯ Very likely
- ◯ Moderately likely
- ◯ Slightly likely
- ◯ Not at all likely
- ◯ Prefer not to answer

* 19. How long will you be willing to participate in the stickers part of the proposed study?

- ◯ Not at all
- ◯ A one off (e.g. for an afternoon or 1 day)
- ◯ Once every now and then (e.g. once a week or once a month)
- ◯ Continuously for a weekend
- ◯ Continuously for a week
- ◯ Continuously for a month
- ◯ Prefer not to answer

* 20. How practical would you find using the above described stickers as a one off (e.g. for one day)?

- ☐ Practical
- ☐ Somewhat practical
- ☐ Somewhat impractical
- ☐ Not at all practical
- ☐ Prefer not to answer
- ☐ Comments

* 21. How practical would you find using the above described stickers over a longer time (e.g. for a week/month)?

- ☐ Practical
- ☐ Somewhat practical
- ☐ Somewhat impractical
- ☐ Not at all practical
- ☐ Prefer not to answer
- ☐ Comments

22. Do you have any comments about the sticker part of the proposed study?

Smart Suits Study

Wrist/ankle bands

Some of the measures of the proposed study will require the infant to wear a wireless wrist/ankle band. This band would be used to measure movement and sleep/wake cycles.
If your youngest child is now older please consider the following questions for when your child was between 3-24 months.

Here is an example of how the wrist/ankle band technology may look.

Image Removed

* 23. How likely would you be to allow your child to wear a wrist or ankle band during the day?

- ◯ Extremely likely
- ◯ Very likely
- ◯ Moderately likely
- ◯ Slightly likely
- ◯ Not at all likely
- ◯ Prefer not to answer

* 24. How likely would you be to allow your child to wear a wrist or ankle band during the night?

- ◯ Extremely likely
- ◯ Very likely
- ◯ Moderately likely
- ◯ Slightly likely
- ◯ Not at all likely
- ◯ Prefer not to answer

* 25. How long will you be willing to participate in the wrist/ankle band part of the proposed study?

- ◯ Not at all
- ◯ A one off (e.g. for an afternoon or 1 day)
- ◯ Once every now and then (e.g. once a week or once a month)
- ◯ Continuously for a weekend
- ◯ Continuously for a week
- ◯ Continuously for a month
- ◯ Prefer not to answer

* 26. How practical would you find having your child wear a wrist or ankle band during the day?

- [ ] Practical
- [ ] Somewhat practical
- [ ] Somewhat impractical
- [ ] Not at all practical
- [ ] Prefer not to answer
- [ ] Comment

* 27. How practical would you find having your child wear a wrist or ankle band during the night?

- ○ Practical
- ○ Somewhat practical
- ○ Somewhat impractical
- ○ Not at all practical
- ○ Prefer not to answer
- ○ Comment

28. Do you have any comments about the wrist/ankle band part of the proposed study?

Smart Suits Study

Body cams

During the proposed study, some of our measures may involve video recording. This will allow us to investigate their behaviour and environment in the home. If your youngest child is now older please consider the following questions for when your child was between 3-24 months.

Here is an example of the size of the proposed body camera technology

Image Removed

* 29. How likely would you be to allow your child to wear a body camera attached to the smart suit (to record your child's environment) during the proposed study?

- ○ Extremely likely
- ○ Very likely
- ○ Moderately likely
- ○ Slightly likely
- ○ Not at all likely
- ○ Prefer not to answer

* 30. How likely would you be to allow a ceiling/cot camera (to analyse your child's behaviour) during the proposed study?

- ○ Extremely likely
- ○ Very likely
- ○ Moderately likely
- ○ Slightly likely
- ○ Not at all likely
- ○ Prefer not to answer

* 31. How long will you be willing to participate in the camera part of the proposed study?

- ○ Not at all
- ○ A one off (e.g. for an afternoon or 1 day)
- ○ Once every now and then (e.g. once a week or once a month)
- ○ Continuously for a weekend
- ○ Continuously for a week
- ○ Continuously for a month
- ○ Prefer not to answer

* 32. When would you be willing to allow us to record?

- During the day
- Day and night
- During the night
- Never
- Prefer not to say
- Specific time (e.g. during play time/afternoon/morning)

Alternatively, in our proposed study, we can use camera technology which automatically analyses the video, such as the number of faces the child sees and the luminance levels. Therefore, the only data our research team will see is an output of numbers, as opposed to actual video recordings.

* 33. If we were to automatically analyse video data (as described above) how likely would you be to allow your child to wear a body camera attached to the smart suit (to analyse your child's environment) during the proposed study?

- Extremely likely
- Very likely
- Moderately likely
- Slightly likely
- Not at all likely
- Prefer not to answer

* 34. If we were to automatically analyse video data (as described above), how likely would you be to allow a ceiling/cot camera (to analyse your child's behaviour) during the proposed study?

- Extremely likely
- Very likely
- Moderately likely
- Slightly likely
- Not at all likely
- Prefer not to answer

* 35. How long will you be willing to participate in the automatically analysed camera part of the proposed study?

- ◯ Not at all
- ◯ A one off (e.g. for an afternoon or 1 day)
- ◯ Once every now and then (e.g. once a week or once a month)
- ◯ Continuously for a weekend
- ◯ Continuously for a week
- ◯ Continuously for a month
- ◯ Prefer not to answer

* 36. How practical do you think it would be to participate in the camera part of the study would be as a one off (e.g. a day)?

- ◯ Practical
- ◯ Somewhat practical
- ◯ Somewhat impractical
- ◯ Not at all practical
- ◯ Prefer not to answer

* 37. How practical do you think it would be to participate in the camera part of the study over an extended period of time (e.g. continuously for a week/month)?

- ☐ Practical
- ☐ Somewhat practical
- ☐ Somewhat impractical
- ☐ Not at all practical
- ☐ Prefer not to answer

* 38. When would you be willing to allow us to record?

◯ During the day ◯ Day and night ◯ During the night ◯ Never ◯ Prefer not to say

◯ Specific time (e.g. during play time/afternoon/morning)

[ ]

39. Do you have any comments about the camera part of the proposed study?

[ ]

Audio recording devices

During the proposed study, some of our measures may require the infants to wear small wireless audio devices. This is so we can measure vocalisations in the home. If your youngest child is now older please consider the following questions for when your child was between 3-24 months.

40. How likely would you be to allow your child to wear an audio recording device?

- ○ Extremely likely
- ○ Very likely
- ○ Moderately likely
- ○ Slightly likely
- ○ Not at all likely

* 41. How long will you be willing to participate in the audio recording part of the proposed study?

- ○ Not at all
- ○ A one off (e.g. for an afternoon or 1 day)
- ○ Once every now and then (e.g. once a week or once a month)
- ○ Continuously for a weekend
- ○ Continuously for a week
- ○ Continuously for a month
- ○ Prefer not to answer

42. How long would you be willing to allow us to record for per day?

- ○ Whole day ○ Day ○ Night ○ Never
- ○ Specific time (e.g. during play time/afternoon/morning)

Alternatively, in our proposed study, we can use technology which automatically analyses the audio recordings, such pitch of voice and number of vocalisations. Therefore, the only data our research team will see is an output of numbers, as opposed to actual audio recordings.

43. If we were to automatically analyses the audio recordings, how likely would you be to allow your child to wear an audio recording device?

- ◯ Extremely likely
- ◯ Very likely
- ◯ Moderately likely
- ◯ Slightly likely
- ◯ Not at all likely

* 44. How long will you be willing to participate in the automatically analysed audio recording part of the proposed study?

- ◯ Not at all
- ◯ A one off (e.g. for an afternoon or 1 day)
- ◯ Once every now and then (e.g. once a week or once a month)
- ◯ Continuously for a weekend
- ◯ Continuously for a week
- ◯ Continuously for a month
- ◯ Prefer not to answer

45. How long would you be willing to allow us to record for per day?

- ◯ Whole day ◯ Day ◯ Night ◯ Never
- ◯ Specific time (e.g. during play time/afternoon/morning)

[ ]

46. How practical do you think the above described audio recording device would be as a one off (e.g. a day)?

- ◯ Practical
- ◯ Somewhat practical
- ◯ Somewhat impractical
- ◯ Not at all practical

47. How practical do you think the above described audio recording device would be for an extended period of time (e.g. a week/month)?

- ◯ Practical
- ◯ Somewhat practical
- ◯ Somewhat impractical
- ◯ Not at all practical

48. Do you have any comments about the audio recording part of the proposed study?

Smart Suits Study

Smart phones

We are proposing to introduce smartphone technology into this new study. During the proposed study, each family will be provided with a smart phone/app with which they can communicate with the technology. The smartphone/app can provide feedback from the data collection, prompts (to answer questionnaires at certain times of the day) and questions about what the infant is doing during the day.

49. How likely would it be for you to use the above described smart phones/app?

- ☐ Extremely likely
- ☐ Very likely
- ☐ Moderately likely
- ☐ Slightly likely
- ☐ Not at all likely
- ☐ Comment

50. How practical would you find using a smart phone/app during the study as a one off (e.g. a day)?

- ◯ Practical
- ◯ Somewhat practical
- ◯ Somewhat impractical
- ◯ Not at all practical

51. How practical would you find using a smart phone/app during the study for an extended period of time (e.g. a week/month)?

- ◯ Practical
- ◯ Somewhat practical
- ◯ Somewhat impractical
- ◯ Not at all practical

52. If you received a prompt via a smart phone app, when would you be most likely to respond immediately?

- ☐ Morning
- ☐ Afternoon
- ☐ Evening
- ☐ Anytime
- ☐ Never

53. How many times a day would you think would be an acceptable number of prompts?

- ◯ None
- ◯ 1
- ◯ 2
- ◯ 4
- ◯ 6
- ◯ no limit

54. Do you have any comments about the smart phone part of the proposed study?

Smart Suits Study

Collected data

Throughout the proposed study parents will be able to access the data being collected. This may be to delete certain parts of the recordings or to check up on their child's sleeping habits.

55. Which of the following collected data would you like access to view? (you can choose more than one)

- [ ] Heart rate
- [ ] Video recording
- [ ] Sweat response
- [ ] Audio recording
- [ ] Sleep measurements
- [ ] Movement

56. From which of the following would you like to be able to access and have the option to ok/delete collected data before we analyse it? (you can choose more than one)

- [ ] Video recording
- [ ] Audio recording
- [ ] Sweat response
- [ ] Movement
- [ ] Sleep
- [ ] Heart rate
- [ ] None of the above

57. If you were to participate in the proposed study, who would you be willing to have access to the collected data?

	Audio/Video recordings	Heart rate/Sweat response
Just our research team	☐	☐
Research teams in the UK	☐	☐
Research teams across Europe	☐	☐
Research teams across the world	☐	☐
All of the above	☐	☐
None of the above	☐	☐

Smart Suits Study

Comments and Questions

58. Do you have any comments/concerns/feedback on any of the above suggested ideas?

Smart Suits Study

Future Participation

59. How interested would you be in potentially participating in our proposed study?

- ◯ Extremely interested
- ◯ Very interested
- ◯ Moderately interested
- ◯ Slightly interested
- ◯ not at all interested

60. Once developed, would you like us to contact you about participating in our proposed study?

- ◯ Yes
- ◯ No
- ◯ n/a (my child is older than 24 months)

If you wish to be contacted about participating in our proposed study please email Laurel Fish on ublfis002@mail.bbk.ac.uk.

Smart Suits Study

Submitting the Survey

Thank you for completing the survey. If you are happy with your answers please press submit. Alternatively you can click previous to go back through or you can click cancel to withdraw your submission.

61. Would you like to submit your answers?

- ◯ Submit
- ◯ Cancel

End of Survey

Thanks for completing the survey. We will share results through the Birkbeck Babylab newsletter.
