# Supplementary material for: A survey on the attitudes of parents with young children on in-home monitoring technologies and study designs for infant research
Source: PLoS One. 2021 Feb 5;16(2):e0245793. doi: 10.1371/journal.pone.0245793 (PMC7864397; doi:10.1371/journal.pone.0245793)
Supplement: S2 File — (DOCX) [file pone.0245793.s002.docx]

S2 File

**A survey on the attitudes of parents with young children on in-home monitoring technologies and study designs for infant research.**

**S2 File**

Table of Contents

[S2: Attrition rates 3](#_Toc61372687)

[S2 Table 1: Attrition per question 4](#_Toc61372688)

[S2 Fig 1: Graphic of attrition 5](#_Toc61372689)

[S3 Table 1: Summary and description of technologies discussed. 7](#_Toc61372690)

[S4: Leximancer Methodology 8](#_Toc61372691)

[S5: Wrist/Ankle band comparisons 16](#_Toc61372692)

[Methods 16](#_Toc61372693)

[Results 16](#_Toc61372694)

[S5: Table summaries of Post Hoc Analysis for sensing wrist bands 21](#_Toc61372695)

[S5 Table 1: Dunn tests for likelihood FANOVA 21](#_Toc61372696)

[S5 Table 2: Binomial tests for band technology participation length 22](#_Toc61372697)

[S5 Table 4: McNemar tests for preferred length of participation between smart suits and wrist/ankle bands 24](#_Toc61372698)

[S6: Additional demographics 25](#_Toc61372699)

[S6 Table 1: Family disorders 25](#_Toc61372700)

[S6 Table 2: Primary caregiver per day 25](#_Toc61372701)

[S7: Table summaries of Post Hoc Analysis for smart suits and stickers 26](#_Toc61372702)

[S7 Table 1: Dun tests for FANOVA on practicality 26](#_Toc61372703)

[S7 Note A: Comparison of length of participation between smart suits and stickers 27](#_Toc61372704)

[S7 Table 2: Binominal tests for Chi-Square test for length of smart suit participation 27](#_Toc61372705)

[S7 Table 3: Binominal tests for Chi-Square test for length of sticker electrodes participation 28](#_Toc61372706)

[S7 Table 4: McNemar tests for Bhapkar test on length of participation between smart suits and sticker electrodes. 29](#_Toc61372707)

[S8: Table summaries of Post Hoc Analysis for smartphones 31](#_Toc61372708)

[S8 Table 1: Dunn tests for Cochran’s test on preferred response time 31](#_Toc61372709)

[S8 Table 2: Binominal tests for Chi-Square test on preferred number of prompts 32](#_Toc61372710)

[S9: Table summaries of Post Hoc Analysis for Privacy 33](#_Toc61372711)

[S9 Table 1: Dunn tests for FANOVA test on video versus audio recording likelihood 33](#_Toc61372712)

[S9 Table 2: Dunn tests for FANOVA test on video/audio recording practicality 34](#_Toc61372713)

[S9 Note A. Comparison of preferred length of participation. 35](#_Toc61372714)

[S9 Table 3: Binominal tests for Chi-Square test on preferred length of manual processed video recording 37](#_Toc61372715)

[S9 Table 4: Binominal tests for Chi-Square test on preferred length of automatic processed video recording 38](#_Toc61372716)

[S9 Table 5: Binominal tests for Chi-Square test on preferred length of manual processed audio recording 39](#_Toc61372717)

[S9 Table 6: Binominal tests for Chi-Square test on preferred length of automatic processed audio recording 40](#_Toc61372718)

[S9 Table 7: McNemar tests for Bhapkar test on preferred length of manual v automatic processed video recording 41](#_Toc61372719)

[S9 Table 8: McNemar tests for Bhapkar test on preferred length of manual v automatic processed audio recording 42](#_Toc61372720)

[S9 Table 9: McNemar tests for Bhapkar test on preferred length of manual processed video v audio recording 43](#_Toc61372721)

[S9 Table 10: McNemar tests for Bhapkar test on preferred length of automatic processed video v audio recording 44](#_Toc61372722)

[S9 Note B: Comparison of when willing to participate per day. 45](#_Toc61372723)

[S9 Table 11: Binomial tests for Chi-Square on preferred time per day of participation with manually processed video recording. 47](#_Toc61372724)

[S9 Table 12: Binomial tests for Chi-Square on preferred length per day with automatically processed video recording. 48](#_Toc61372725)

[S9 Table 13: Binomial tests for Chi-Square on preferred length per day with manually processed audio recording. 49](#_Toc61372726)

[S9 Table 14: Binomial tests for Chi-Square on preferred length per day with automatically processed audio recording. 50](#_Toc61372727)

[S9 Table 15: McNemar tests for Bhapkar test on when willing to participate per day between manually processed and automatically processed video recording. 51](#_Toc61372728)

[S9 Table 16: McNemar tests for Bhapkar test on when willing to participate per day between manually processed and automatically processed audio recording. 52](#_Toc61372729)

[S9 Table 17: McNemar tests for Bhapkar test on when willing to participate per between manually processed video and audio recording. 53](#_Toc61372730)

[S10: Table summaries of Post Hoc Analysis for Data Sharing 54](#_Toc61372731)

[S10 Table 1: Binomial tests for Chi-Square on preferred data sharing for anonymous data. 54](#_Toc61372732)

[S10 Table 2: Binomial tests for Chi-Square on preferred data sharing for non-anonymous data. 55](#_Toc61372733)

[S10 Table 3: McNemar tests for Bhapkar on preferred data sharing between anonymous and non-anonymous. 56](#_Toc61372734)

[S11: Table summaries of Post Hoc Analysis for Data access 57](#_Toc61372735)

[S11 Table 1: Dunn tests for Cochran’s test on viewing each type of data. 57](#_Toc61372736)

[S11 Table 2: Dunn tests for Cochran’s test on ok/deleting each type of data. 58](#_Toc61372737)

[S12: Table summaries of Post Hoc Analysis for Future Participation 59](#_Toc61372738)

[S12 Table 1: Dunn Test for Chi-Square on interest for future participation 59](#_Toc61372739)

[S12 Table 2: Dun Test for Chi-Square on interest for future participation 60](#_Toc61372740)

[S12 Note A: Reuse of smart suits Analysis. 60](#_Toc61372741)

[S13: Low SES Subset Analysis 61](#_Toc61372742)

[Practicability 61](#_Toc61372743)

[Study interaction 62](#_Toc61372744)

[Privacy 62](#_Toc61372745)

[Data sharing 63](#_Toc61372746)

[S13: Table summaries of Post Hoc Analysis of Subset Analysis 66](#_Toc61372747)

[S13 Table 1: Dunn tests FANOVA practicality smart suits and sticker electrodes. 66](#_Toc61372748)

[S13 Table 2: Dunn tests Cochran’s test for viewing each type of data. 67](#_Toc61372749)

[S13 Table 3: Dunn tests Cochran’s test for OK/Delete each type of data. 68](#_Toc61372750)

[S14 Note A 70](#_Toc61372751)

# S2: Attrition rates

## S2 Table 1: Attrition per question

Attrition rates calculated based on the 410 participants who completed at least one question after the initial consenting question (Q1).

| Survey Question number | Participants Included  (n) | Participants Skipped  (n) | Attrition Rate (%) | Prefer not to answer  (n) |
| --- | --- | --- | --- | --- |
| Q1 | 410 | 0 | 0 | 0 |
| Q2 | 410 | 0 | 0 | 0 |
| Q3 | 410 | 1 | 0.24 | 0 |
| Q4 | 410 | 0 | 0 | 2 |
| Q5 | 410 | 0 | 0 | 0 |
| Q6 | 410 | 0 | 0 | 3 |
| Q7 | 410 | 0 | 0 | 0 |
| Q8 | 410 | 9 | 2.20 | 19 |
| Q9 | 410 | 9 | 2.20 | 0 |
| Q10 | 410 | 9 | 2.20 | 1 |
| Q11 | 410 | 9 | 2. 20 | 2 |
| Q12 | 410 | 24 | 5.85 | 0 |
| Q13 | 410 | 24 | 5. 85 | 4 |
| Q14 | 410 | 24 | 5. 85 | 0 |
| Q15 | 410 | 25 | 6.10 | 0 |
| Q16 | 410 | 24 | 5. 85 | 9 |
| Q17 | 410 | 251 | 61.22 | N/A |
| Q18 | 410 | 32 | 7.80 | 0 |
| Q19 | 410 | 32 | 7.80 | 3 |
| Q20 | 410 | 32 | 7.80 | 3 |
| Q21 | 410 | 32 | 7.80 | 2 |
| Q22 | 410 | 259 | 63.17 | N/A |
| Q23 | 410 | 36 | 8.78 | 2 |
| Q24 | 410 | 36 | 8.78 | 2 |
| Q25 | 410 | 36 | 8.78 | 4 |
| Q26 | 410 | 36 | 8.78 | 2 |
| Q27 | 410 | 36 | 8.78 | 1 |
| Q28 | 410 | 304 | 74.15 | N/A |
| Q29 | 410 | 47 | 11.46 | 2 |
| Q30 | 410 | 47 | 11.46 | 3 |
| Q31 | 410 | 47 | 11.46 | 4 |
| Q32 | 410 | 47 | 11.46 | 5 |
| Q33 | 410 | 47 | 11.46 | 0 |
| Q34 | 410 | 47 | 11.46 | 0 |
| Q35 | 410 | 47 | 11.46 | 2 |
| Q36 | 410 | 47 | 11.46 | 3 |
| Q37 | 410 | 47 | 11.46 | 3 |
| Q38 | 410 | 47 | 11.46 | 8 |
| Q39 | 410 | 306 | 74.63 | N/A |
| Q40 | 410 | 66 | 16.10 | 0 |
| Q41 | 410 | 64 | 15.61 | 2 |
| Q42 | 410 | 64 | 15.61 | 0 |
| Q43 | 410 | 64 | 15.61 | 0 |
| Q44 | 410 | 64 | 15.61 | 2 |
| Q45 | 410 | 68 | 16.59 | 0 |
| Q46 | 410 | 67 | 16.34 | 0 |
| Q47 | 410 | 66 | 16.10 | 0 |
| Q48 | 410 | 341 | 83.17 | N/A |
| Q49 | 410 | 72 | 17.56 | 0 |
| Q50 | 410 | 73 | 17.80 | 0 |
| Q51 | 410 | 72 | 17.56 | 0 |
| Q52 | 410 | 73 | 17.80 | 0 |
| Q53 | 410 | 73 | 17.80 | 0 |
| Q54 | 410 | 354 | 86.34 | N/A |
| Q55 | 410 | 79 | 19.27 | 0 |
| Q56 | 410 | 79 | 19.27 | 0 |
| Q57 | 410 | 76 | 18.54 | 0 |
| Q58 | 410 | 316 | 77.07 | N/A |
| Q59 | 410 | 78 | 19.02 | 0 |
| Q60 | 410 | 76 | 18.54 | 0 |
| Q61 | 410 | 77 | 18.79 | 0 |

*Note*. Questions marked in grey are those that were completely optional and did not require an answer to continue to the next question. Attrition rate is calculated to include those who responded “prefer not to answer”.

## S2 Fig 1: Graphic of attrition

*S2 Fig 1.* Attrition rate per question of the questionnaire. Question 17, 22, 28, 39, 48, 54, 58 are excluded from the graph as these questions were completely optional, hence have a considerable higher attrition than the rest of the questionnaire and do not reflect the overall attrition rate.

# S3 Table 1: Summary and description of technologies discussed.

| Technology | Description |
| --- | --- |
| Smart suits | Typical babygrow or romper suit made with “smart” textiles consisting of smart sensing fibres that are in contact with the child’s skin. These suits can detect a range of physiological responses (e.g. skin conductance and heart rate) and movement. |
| Sensing bands | A wrist or ankle band sensors that are in contact with child’s skin. These bands can detect a range of physiological responses (e.g. skin conductance and heart rate) and movement. |
| sticker sensing electrodes | Sensing electrodes that are placed directly onto the child’s skin and are secured using adhesive stickers. These sensing electrodes are connected via wires to a small device to be carried in the infant’s pocket. The electrodes can measure a range of physiological responses (e.g. skin conductance and heart rate) and movement. |
| Smart phones applications | Application that are downloadable to smart phone that enable either self-report primary data recording, prompts for other device, or study interactions (e.g. to communicate with the researcher’s information about current activity or environment). |
| Audio devices | Audio recording devices that can record sound in the environment. |
| On-body camera | Mobile cameras that can be attached to the child that will record the child’s environment. |
| Cot camera | Static cameras that are attached to a cot or a room that will record the child’s environment and behaviour. |

# S4: Leximancer Methodology

We used Leximancer Desktop 5.0 (1) to generate concepts and themes from open field general comment boxes. Leximancer Desktop 5.0 is an unsupervised text-mining software. This software is validated (2) and has been used in a number of primary research articles to explore qualitative data (3–5), including analysis of stakeholder opinions (6). Leximancer Desktop 5.0 automatically extrapolates concepts by grouping terms with similar meaning and relational connections (i.e. proximity within text experts of two sentences). Concept co-occurrence correlations are derived, to aid further grouping into overarching “Themes”. Extracted concepts and themes are displayed in topographical network maps. “Themes” are depicted as coloured circles of differing sizes (larger the circle the more prevalent the concept) and “concepts” are grey dots. Topographic proximity of objects in the map indicates contextual interrelatedness.

Leximancer has a built in stopword list of predefined words not included in concept generation as they are not useful for developing clearly-define concepts (e.g. “and”). We additionally removed or merged together concepts depending on whether we deemed them useful for generating concepts that would enable further understanding of stakeholder’s opinions on each technology. For example, for the general comments on camera technology, having a concept of “camera” would be useful as the entire map is derived from opinions on camera usage. Including “camera” as a concept condenses smaller, more specific concepts into one large theme, preventing extrapolation of more informative concepts. See S4 Fig 1-7 for a full list of removed and merged concepts depicted within the *Concept Editing* boxes alongside topographic theme/concept plots with and without stop word supervision for each general comment question.

**Smart Suits**

a. Theme and concept map **before** concept editing


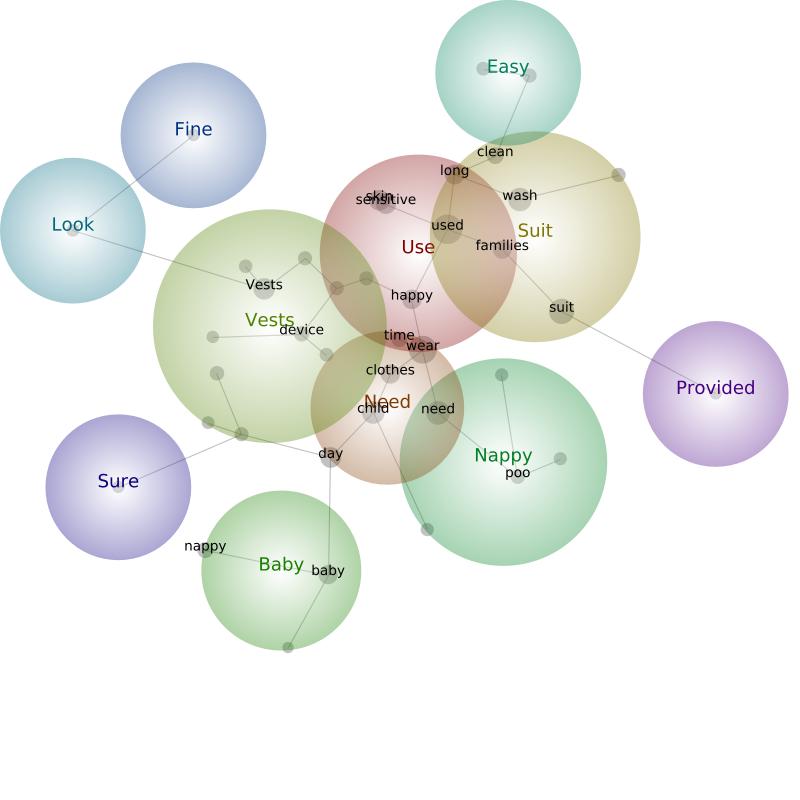


b. **Concept Edits**

*Removed:*

- Suits/Vest/Baby/Child/Daughter/Use/Wear/Try
  - The question asked about general comments on *using suits/vest devices on child* therefore including these words would not add any value to the analysis of stakeholder’s opinions.
- Sure/Due
  - Words with low semantic-content therefore added to the *stopword list*

*Merged*

- Wash/Clean/Nappy/Poo/Stains = Hygiene
- Technology/Device = Technology
- Material/Cotton = Material

c. Theme and concept map **after** concept

Editing


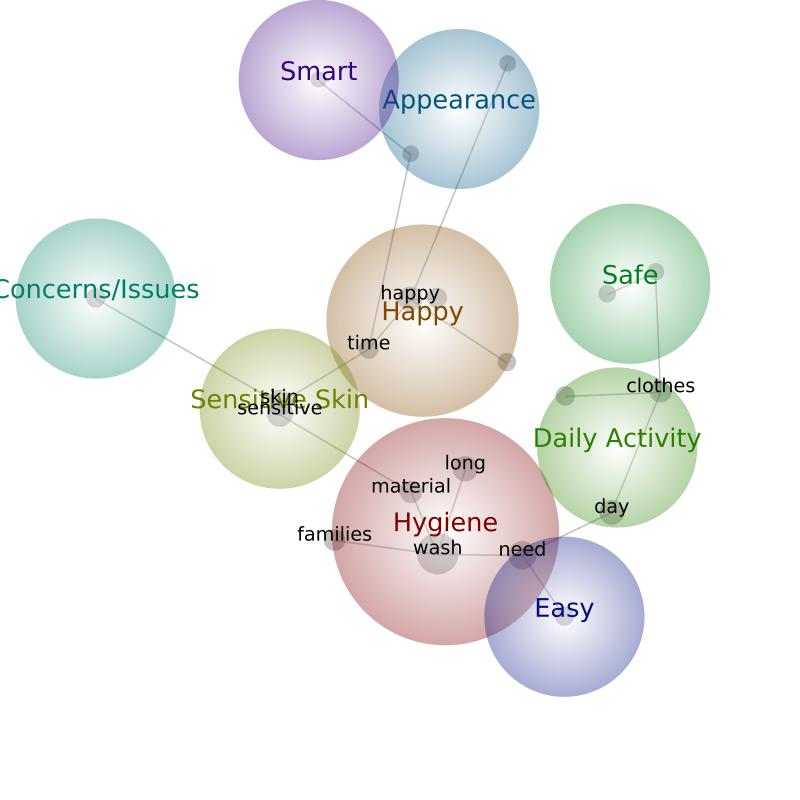


S4 Fig 1. Generation of topographic concept/theme maps for open field general comment for smart suits. a) Theme and concepts maps before concept editing. b) Concept Editing information, explaining those concepts what were removed and why, and those that were merged. c) Theme and concepts maps after concept editing.

**Sticker Electrodes**

a. Theme and concept map **before** concept editing
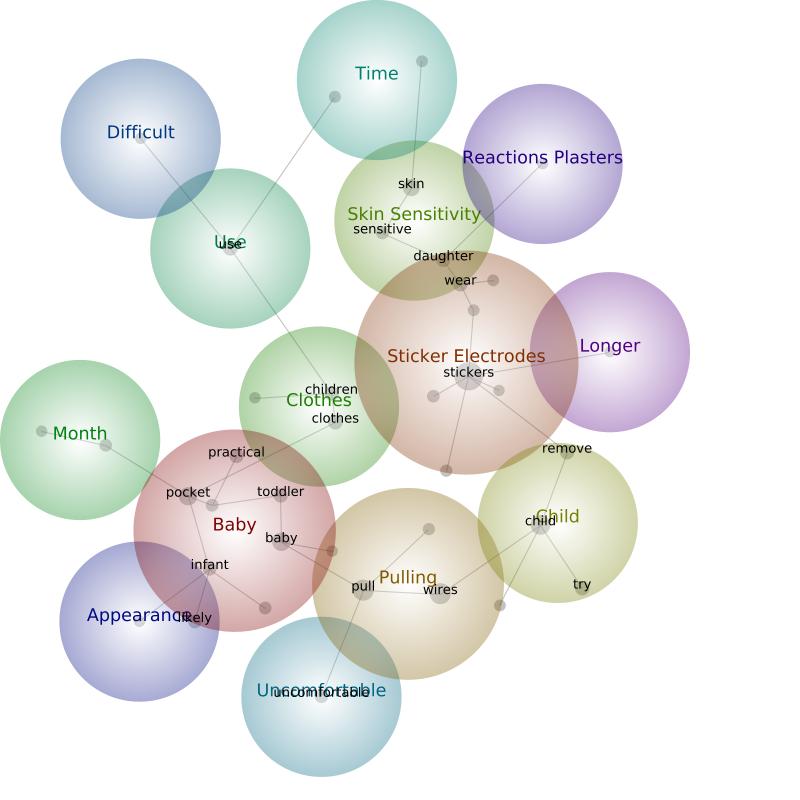


c. Theme and concept map **after** concept

editing


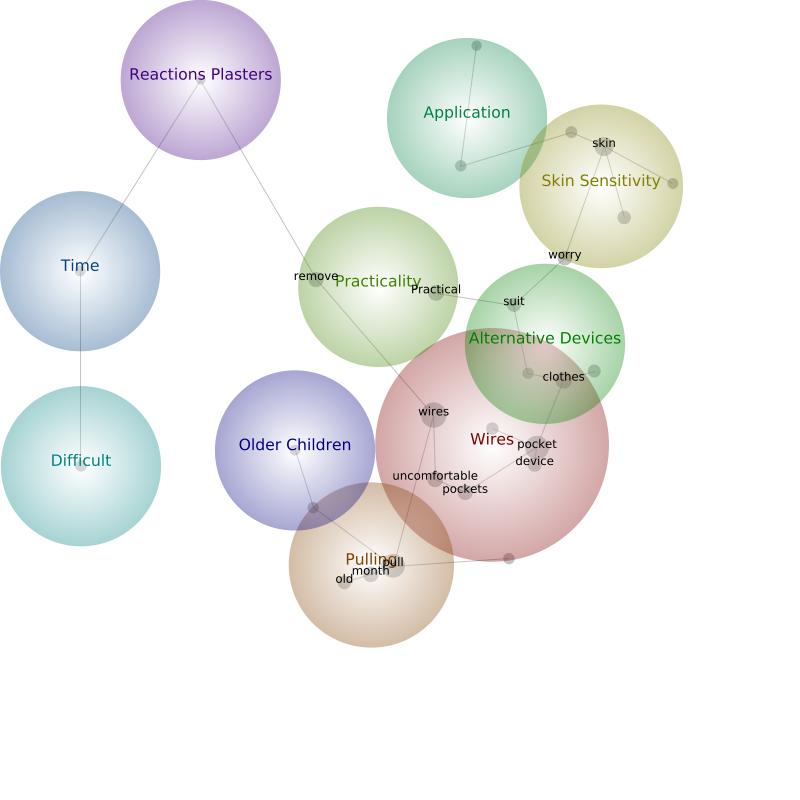


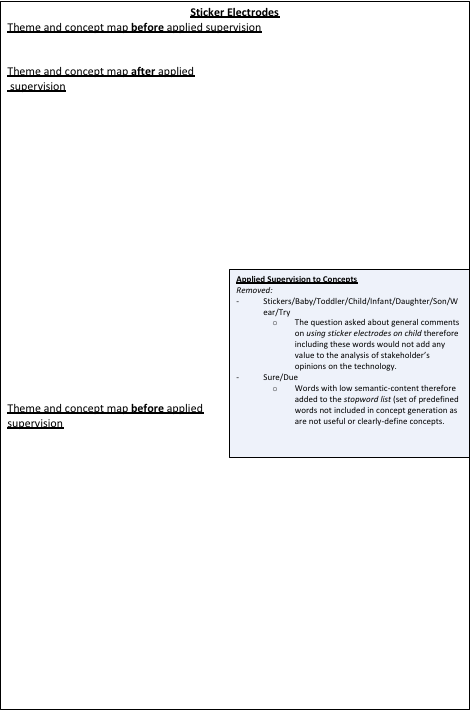


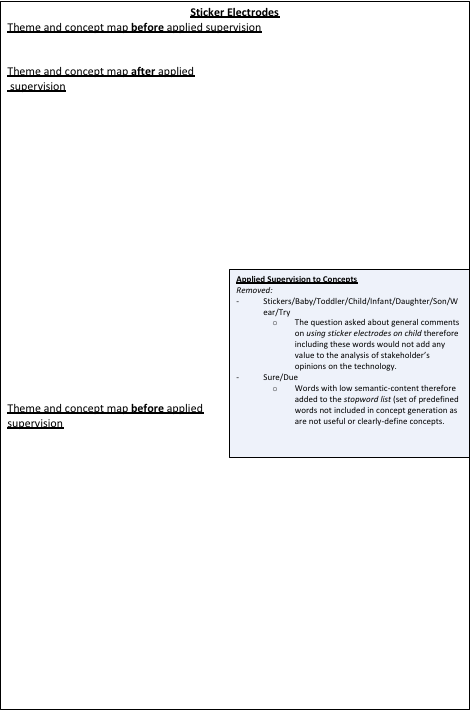


Theme and concept map **before** applied

supervision

b. **Concept Edits**

*Removed:*

- Stickers/Baby/Toddler/Child/Infant/Daughter/Son/Wear/Try
  - The question asked about general comments on *using sticker electrodes on child* therefore including these words would not add any value to the analysis of stakeholder’s opinions on the technology.
- Sure/Due
  - Words with low semantic content therefore added to the *stopword list.*

S4 Fig 2. Generation of topographic concept/theme maps for open field general comment for sticker electrodes. a) Theme and concepts maps before concept editing. b) Concept Editing information, explaining those concepts what were removed and why, and those that were merged. c) Theme and concepts maps after concept editing.

**Bands**

a. Theme and concept map **before** concept editing


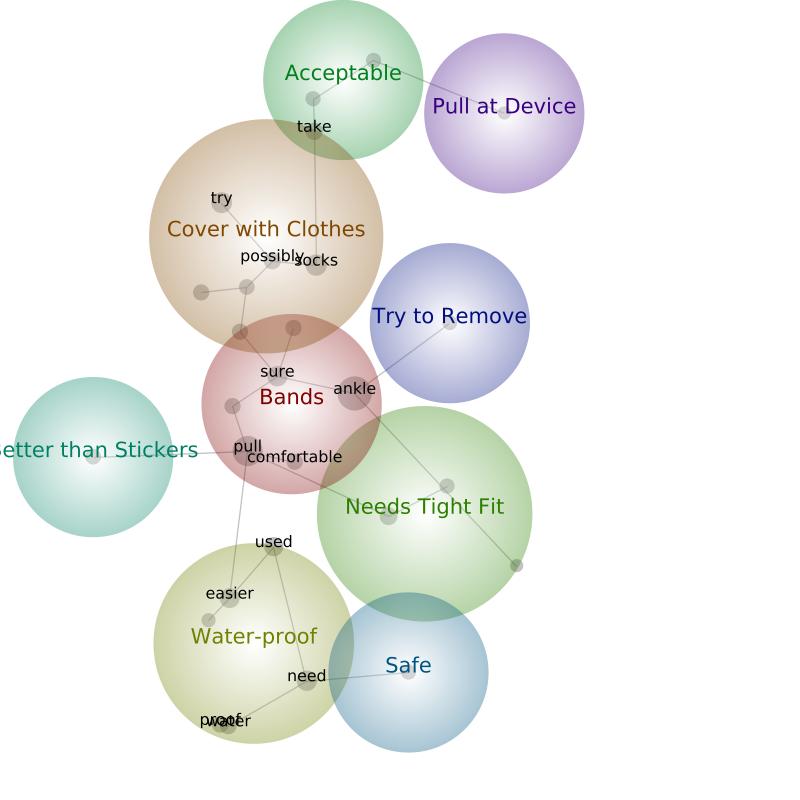


c. Theme and concept map **after** concept

Editing


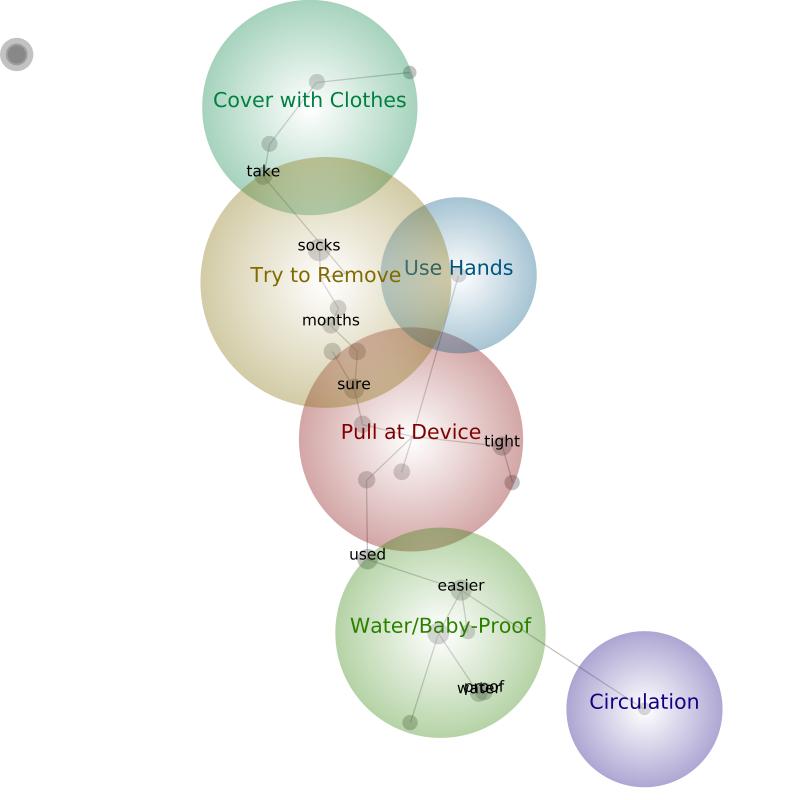


b. **Concept Editing**

*Removed:*

- Bands/Ankle/Wrist/Baby/Toddler/Child/Son
  - The question asked about general comments on *using sticker electrodes on child* therefore including these words would not add any value to the analysis of stakeholder’s opinions on the technology.

S4 Fig 3. Generation of topographic concept/theme maps for open field general comment for sensing bands. a) Theme and concepts maps before concept editing. b) Concept Editing information, explaining those concepts what were removed and why, and those that were merged. c) Theme and concepts maps after concept editing.

**Camera**

a. Theme and concept map **before** concept editing


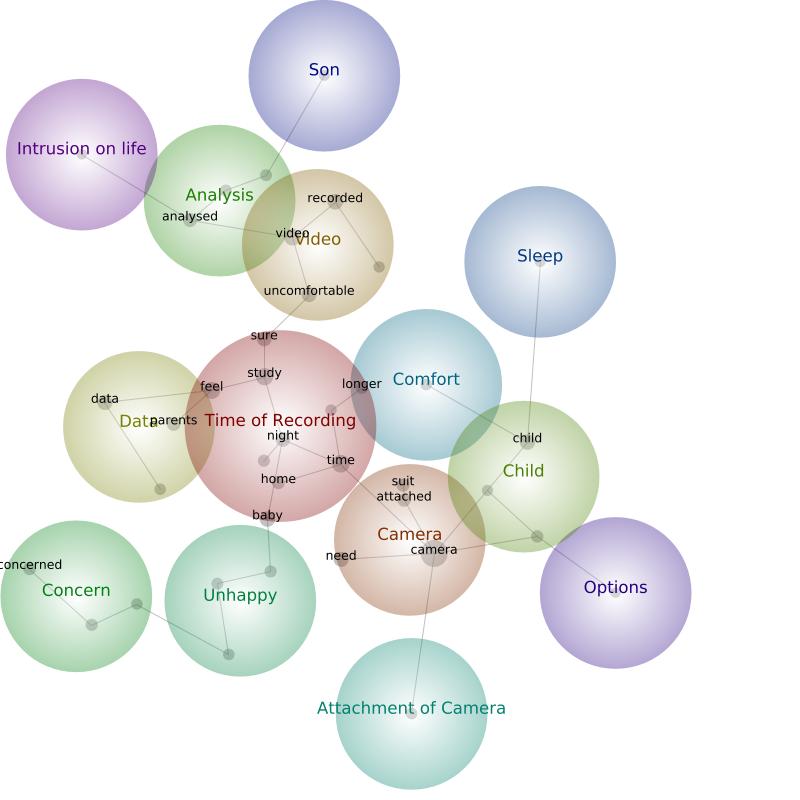


c. Theme and concept map **after** concept

editing


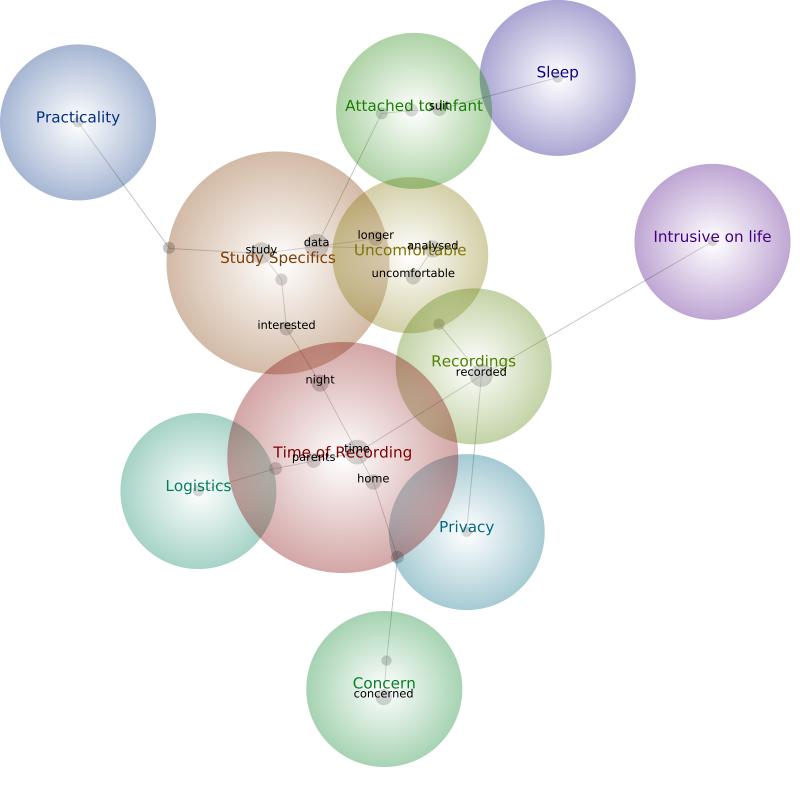


b. **Concept Editing**

*Removed:*

- Baby/Toddler/Child/Son/Filming/Video/Camera
  - The question asked about general comments on *using sticker electrodes on child* therefore including these words would not add any value to the analysis of stakeholder’s opinions on the technology.
- Due/Sure/feel
  - Words with low semantic content therefore added to the *stopword list*.

*Merged*

- Worry/concern = Worry
- Researcher/Study = Study
- Footage/data = data

S4 Fig 4. Generation of topographic concept/theme maps for open field general comment for camera recording. a) Theme and concepts maps before concept editing. b) Concept Editing information, explaining those concepts what were removed and why, and those that were merged. c) Theme and concepts maps after concept editing.

**Audio**

a. Theme and concept map **before** concept editing


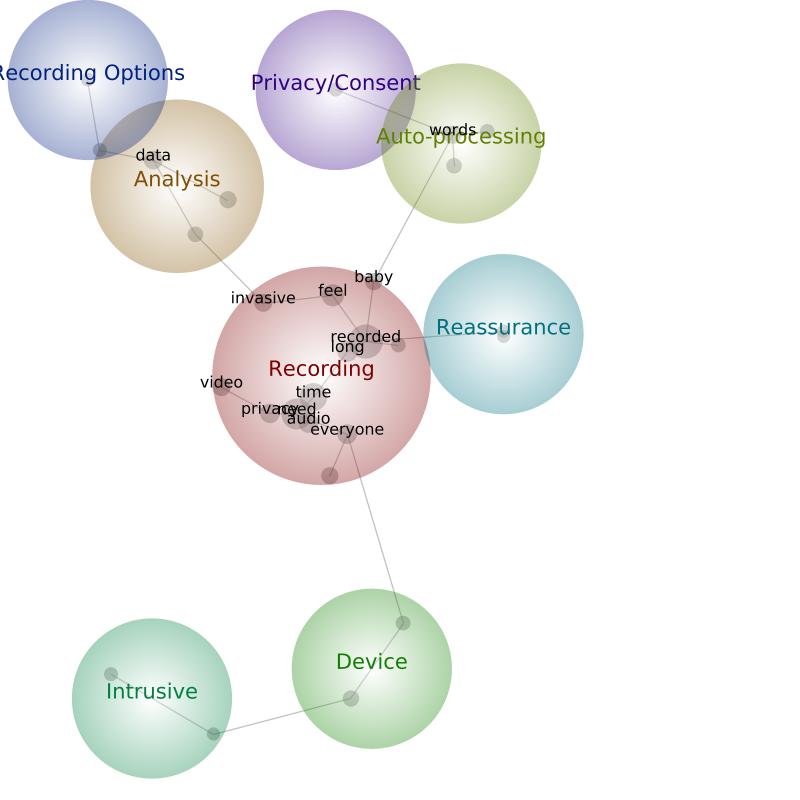


c. Theme and concept map **after** concept editing


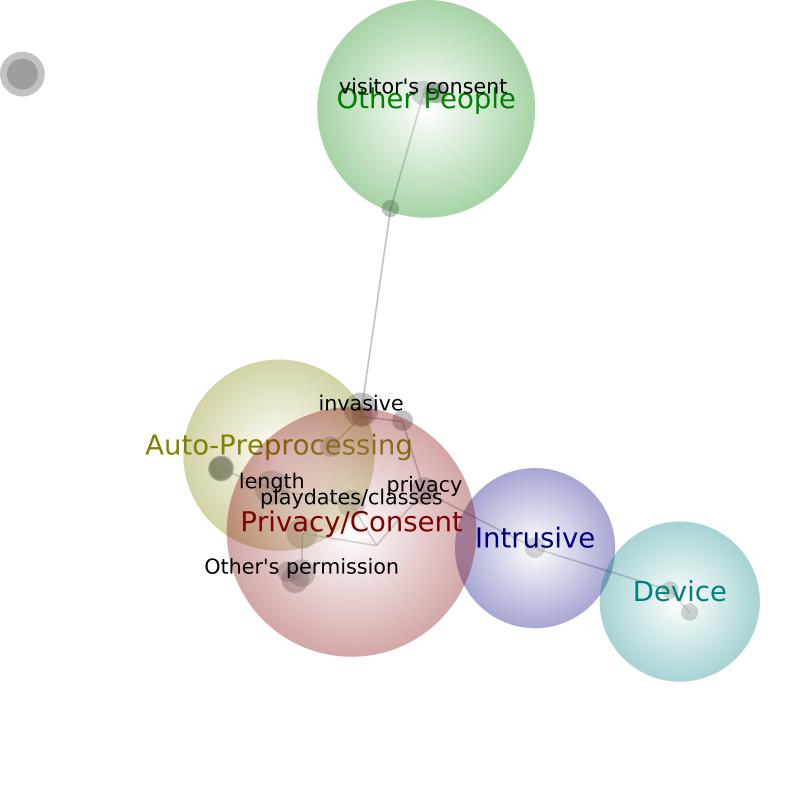


b. **Concept Editing**

*Removed:*

- Baby/Toddler/Child/Son/Use/Audio/Recorded
  - The question asked about general comments on *using sticker electrodes on child* therefore including these words would not add any value to the analysis of stakeholder’s opinions on the technology.
- Having/ feel
  - Words with low semantic content therefore added to the *stopword list*

S4 Fig 5. Generation of topographic concept/theme maps for open field general comment for audio recording. a) Theme and concepts maps before concept editing. b) Concept Editing information, explaining those concepts what were removed and why, and those that were merged. c) Theme and concepts maps after concept editing.

**Smart Phones**

a. Theme and concept map **before** concept editing


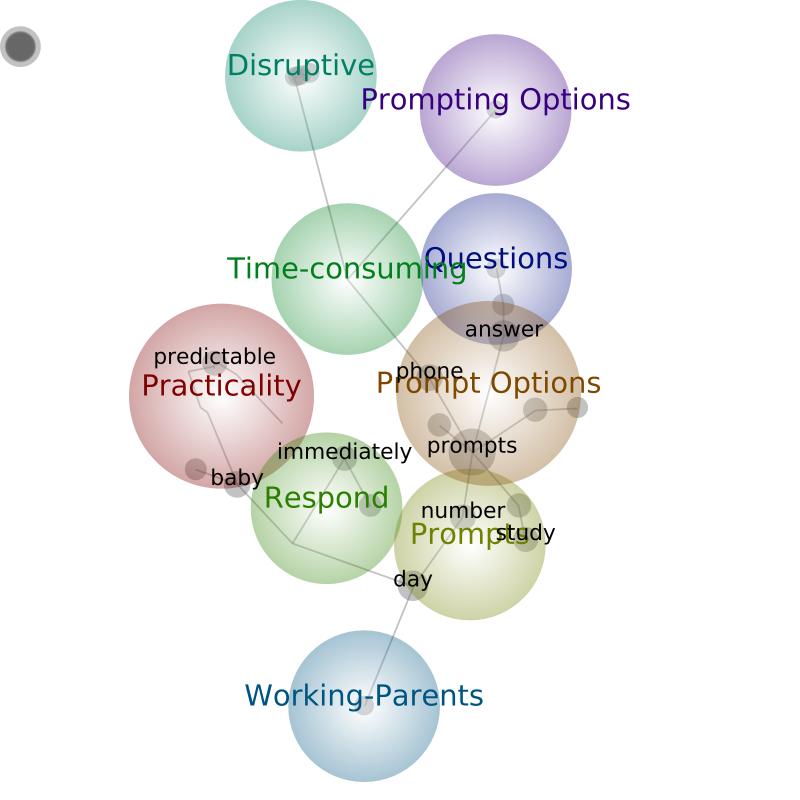


c. Theme and concept map **after** concept editing


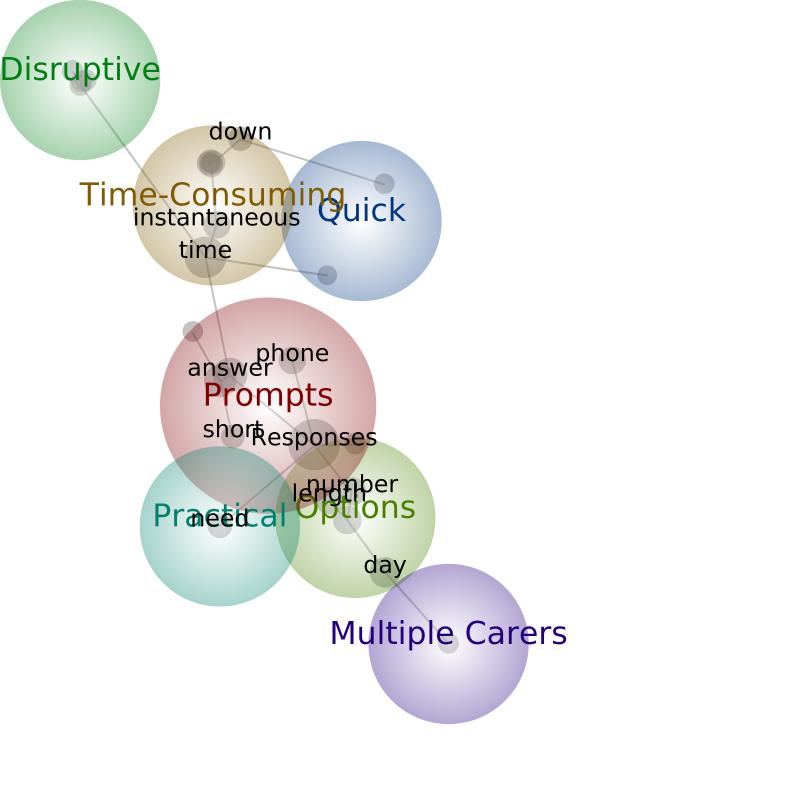
­

b. **Concept Editing**

*Removed:*

- Baby/Child
  - The question asked about general comments on *using sticker electrodes on child* therefore including these words would not add any value to the analysis of stakeholder’s opinions on the technology.
- Do/Doing
  - Words with low semantic-content therefore added to the *stopword list*

*Merged*

- *Questions/Questionnaires = Questions*
- *Respond/Response = Response*
- *Immediate/Instantaneous = Immediate*

S4 Fig 6. Generation of topographic concept/theme maps for open field general comment for smartphones. a) Theme and concepts maps before concept editing. b) Concept Editing information, explaining those concepts what were removed and why, and those that were merged. c) Theme and concepts maps after concept editing.

**General**

Theme and concept map **without** concept editing


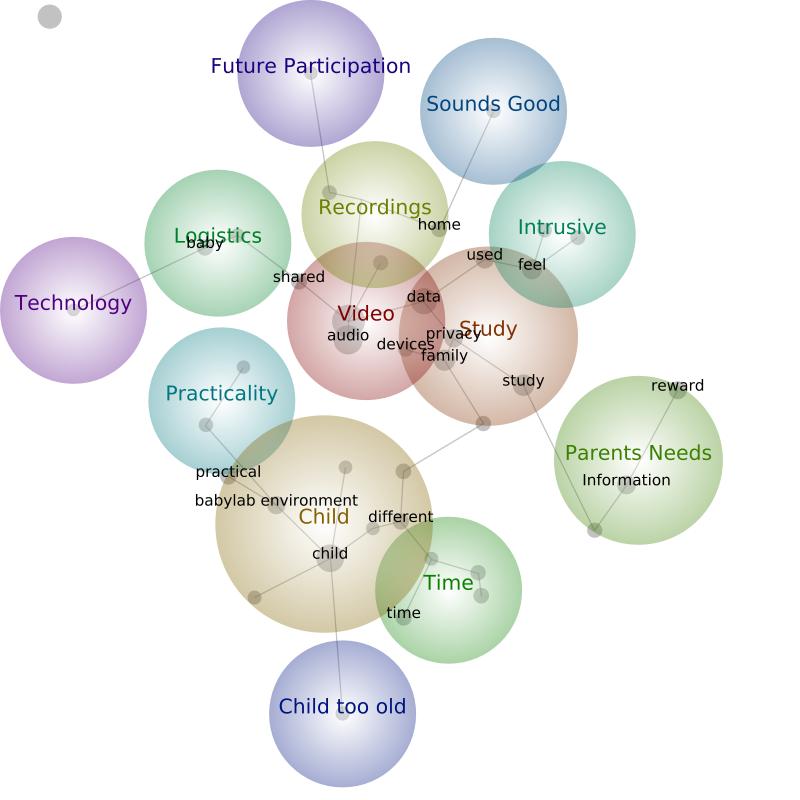


S4 Fig 7. Theme and concepts maps for general comment for question about the study as whole. *Note.* No concept editing was applied in order to capture the wide range of concept and themes provided by participants.

# S5: Wrist/Ankle band comparisons

## **Methods**

***Practicality.*** It is important to gauge multiple aspects of certain devices. As wrist/ankle bands may be slightly easier than other technologies for the infant to remove themselves, we asked whether parents their opinions to practicality and likelihood of participation differed between night and day. Using Wilcoxon Match-Pairs Signed-Ranks Test (WMPSR), we tested the effect of day/night-time on a) likelihood rating b) practicality.

To examine practicality and acceptability of infant wrist/ankle band wearable technologies compared assigned rating for band technology with sticker (electrodes) and smart suits. Using Friedman’s analysis of Variance (FANOVA) with pairwise Dunn-Bonferroni test, we tested the effect of the three technologies on changes to likelihood rating. For each technology, we analysed whether participants were equally likely to accept different recording lengths (i.e. whether responses were evenly distributed across time intervals) using Pearson Chi-square goodness-of-fit with Bonferroni corrected binominal pairwise comparisons. Finally, to investigate whether preferences for participation length differed between technologies, we used Bhapkar tests with 2*2 continency Bonferroni corrected McNemar post hoc test to compare selecting versus not selecting each category between technologies. We also generated a word map to depict general comments made on bands.

## **Results**

**Comparisons with Wrist/Ankle Band ratings**

**Comparison of likelihood.** Overall, median likelihood responses were higher for using bands during the day (Mdn = 5.00, *M* = 4.31, *SD* = 0.86) than at night (*Mdn* = 4.00, *M* = 4.10, *SD* = 1.05), S5 Fig 1A. Wilcox Matched-Pairs Signed-Ranks Test (WMPSR) revealed significantly more participants (n = 79) rated using bands during the day more likely than at night compared to those who rated day time less likely (n = 20), *z* = -5.51, *p* < .001, with low effect size (*r* = -0.29). 261 people rated participation with each technology as equally likely.

**Comparison of practicality.** Overall, median practicality responses were equal for using bands during the day (*Mdn* = 3.00, *M* = 3.15, *SD* = 0.82) than at night (*Mdn* =3.00, *M* = 3.23, *SD* = 0.84), S5 Fig 1b. Wilcox Matched-Pairs Signed-Ranks Test (WMPSR) revealed no significant difference between participant’s practicality rating, *z* = -1.06, *p* = 0.29. Two hundred and forty-seven people rated participation with each technology as equally likely, 43 rated daytime more practical as night, and 53 rated daytime less practical then night.

**Comparisons between Sticker Electrodes, Wrist/Ankle Bands and Smart Suits**

**Comparison of likelihood.** To enable comparisons across multiple technologies, the median responses to the two wrist/ankle bands likelihood questions (i.e. likelihood of using a) at night and b) during the day) was calculated per participant to create an individual’s overall likelihood score for wrist/ankle bands. Responses to likelihood questions are depicted in S5 Fig 1c.

A FANOVA identified an overall significant difference in rating of likelihood between sticker electrode technology (*Mdn* = 3.00, *M* = 2.91, *SD* = 1.32), wrist/ankle bands (*Mdn* = 4.50, *M* = 4.20, *SD* = 0.90), and smart suits (*Mdn* = 5.00, *M* = 4.37, *SD* = 0.79), *χ^2^*(2, n = 370) = 350.02, p < 0.001. Further analysis revealed a Kendall W of .473, indicating a moderate level of agreement among responders. Dunn post-hoc tests with Bonferroni adjustments (See Table 4) revealed significant differences in ratings between wrist/ankle bands and sticker sensing electrode technologies (*p* < 0.001), with wrist/ankle bands rated more likely than sticker sensing electrodes. No significant differences were found between wrist/ankle bands and smart suits (*p* = 0.08).

**Comparison of length of participation.** Parents thought smart suits and wrist bands to be more acceptable for longer periods of time, see S5 Fig 1d. A Pearson’s Chi-Square goodness-of-fit (Chi-Square) test indicated preferences for the length of time choices were not equally distributed for all technologies (refer to main text practicality section page 13 for smart suits and sticker electrode results). Pearson’s chi square results indicate responses for bands to be significantly unevenly distributed to a moderate effect (*χ^2^*(5, *N* = 366) = 180.03, *p* < .001, *Cramer’s V* = 0.31). “Month” was indicated as the most preferred option. Pairwise comparisons with Bonferroni adjusted binomial test (see Table 5) partially confirmed this observation, with “Month” receiving significantly more votes for than all other length of choices except “Week” (*p* = 0.94).

Bhapkar tests revealed technology caused significant changes to individuals preferred recording time between wrist/ankle band technology and: sticker electrode technology, *χ^2^*(5, *N* = 365) = 623, *p* < .001; and smart suits, *χ^2^*(5, *N* = 363) = 11.4, *p* = 0.04. Of the 78% of responders who changed their response between sensing electrode stickers and bands, 98% chose longer periods of study duration for bands. McNemar post-hoc tests with Bonferroni adjustments (See Table 6) revealed proportions of responders changed significantly for “Not at all” (*p* < .001), “Once” (*p* < .001), “Now and Then” (*p* < .001), “Weekend” (*p* < .001) and “Month” (*p* < .001) with responders tending to change their option to longer lengths of time for wrist/ankle bands than sensing electrode stickers. Of the 94%, 90%, 83%, 95% and 80% of responders who changed their response when selecting “Not at all”, “Once”, “Now and Then”, “Week” and “Month” for either bands or sensing electrode stickers, respectively, 98%, 96%, 97%, 99% and 100% chose relatively longer periods of study duration for bands. No significant changes across wrist/ankle band and sensing electrode stickers for “Weekend” (*p* = 0.35).

For comparisons between smart suits and wrist/ankle bands, Of the 63% of responders who changed their response between sensing electrode stickers and bands, 66% chose longer periods of study duration for bands. Pairwise McNemar post-hoc tests with Bonferroni Corrections (See Table 7), revealed proportions of responses changed significantly only for “Month” (*p* = 0.05). Of the 45% of responders who changed their response when selecting “Month” for either bands or sensing electrode stickers 66% chose relatively longer periods of study duration for bands. No other lengths of time indicated to be significantly different between smart suits and wrist/ankle bands (p = 1.00).

­

A.

B.

C.

D.

*S5 Fig 1*. Percentages of response frequency for questions concerning practicality and acceptability of bands compared to smart suits and sticker electrode technologies *A.* Participation likelihood rating for using bands either in the daytime or night time. *B.* Practicality rating for using bands at night or during day. *C.* Participation likelihood rating for bands*, sticker electrodes and smart suits. *D*. Practicality rating for sticker electrodes, wrist/ankle bands and smart suits.

*Note.* Percentages were calculated out of all responders who answered per question, including ‘Prefer not to answer’ responses*.* * band ratings were a median score across day and night questions for overall band likelihood in Fig C. SW = Somewhat


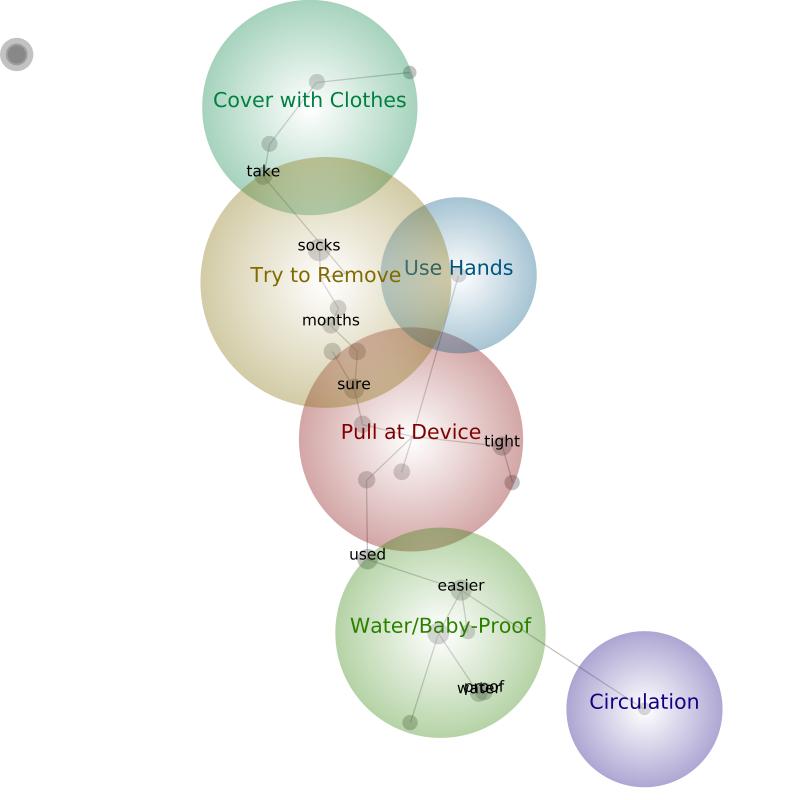
**General comments.** Concept maps depicting the prevalence and co-occurrence of themes and concepts in the general comments question for sensing bands were generated (see S5 Fig 2a-b). As indicated by the number of hits, the device being “Water/Baby-Proof” was the most commented theme.

*S5 Fig 2*. a) Theme/concept maps depicting prevalence and co-occurrence of words from general comments questions regarding sensing wrist/ankle bands. b) Table displaying each theme and its relative hit number.

B.

| **Theme** | **Hits** |
| --- | --- |
| Water/Baby-Proof | 23 |
| Pull at Device | 21 |
| Try to remove | 20 |
| Cover with Clothes | 4 |
| Circulation | 3 |
| Use Hands | 3 |

A.

## S5: Table summaries of Post Hoc Analysis for sensing wrist bands

### S5 Table 1: Dunn tests for likelihood FANOVA

Dunn post-hocs Test for FANOVA on rating regarding likelihood of using smart suits, sticker electrodes or wrist/ankle bands.

| Dunn Test Comparison | |  |  |
| --- | --- | --- | --- |
| Question 1  *(M, SD)* | Question 2  *(M, SD)* | Standardised Test Statistic | Adj. p Value  (Bonf. Corr.) |
| Smart Suits  (4.37, 0.79) | Sticker Electrodes  (2.91, 1.32) | 14.87 | < 0.001*** |
|  | Wrist/Ankle Band  (4.20, 0.90) | 2.22 | 0.08 |
| Sticker Electrodes  (2.91, 1.32) | Wrist/Ankle Band  (4.20, 0.90) | 12.65 | < 0.001*** |

*Note:* Adj. p Value (Bonf. Corr.) = Bonerfoni corrected p value. *** denotes significance.

### S5 Table 2: Binomial tests for band technology participation length

Binomial post hoc tests for Chi-Square test on distribution of response frequency for question regarding preferred length of participation for wrist/ankle band technology.

| Binomial Comparisons | |  |  |
| --- | --- | --- | --- |
| Response 1  (n) | Response 2  (n) | Adj. p Value  (Bonf. Corr.) | Relative Risk* |
| Not at all  (4) | Once  (19) | 0.04*** | 0.35 |
|  | Now and Then  (61) | 5.88 *10^-13^*** | 0.12 |
|  | Weekend  (55) | 2.55 *10^-11^*** | 0.14 |
|  | Week  (99) | 1.36 *10^-23^*** | 0.08 |
|  | Month  (128) | 6.87*10^-32^*** | 0.06 |
| Once  (19) | Now and Then  (61) | 4.10*10^-05^*** | 0.48 |
|  | Weekend  (55) | 5.07*10^-04^*** | 0.51 |
|  | Week  (99) | 4.58 *10^-13^*** | 0.32 |
|  | Month  (128) | 7.25 *10^-20^*** | 0.26 |
| Now and Then  (74) | Weekend  (55) | 9.64 | 1.06 |
|  | Week  (99) | 4.97 *10^-2^ *** | 0.76 |
|  | Month  (128) | 1.86 *10^-6^ *** | 0.65 |
| Weekend  (55) | Week  (99) | 7.34*10^-3^ *** | 0.71 |
|  | Month  (128) | 1.04 *10^-08^*** | 0.60 |
| Week  (99) | Month  (128) | 0.90 | 0.87 |

Note: *Adj. p Value (Bonf. Corr.)* = Bonferroni corrected p value. *Relative risk denotes probability ratio of participant choosing response 1 over response 2. *** denotes significance.

S5 Table 3: McNemar tests for preferred length of participation between sticker electrodes and wrist/ankle bands

McNemar post hoc tests for Bhapkar test on response frequency for question regarding preferred length of participation between sticker electrodes and wrist/ankle bands.

| Response Option  (n = a, b, c, d) | McNemar *χ^2^* | Adj. p Value  (Bonf. Corr.) |
| --- | --- | --- |
| Not at all  (3, 44, 1, 317) | 41.09 | 8.73 *10^-10^*** |
| Once  (13, 107, 6, 239) | 90.27 | 1.24 *10^-20^*** |
| Now and Then  (25, 90, 35, 215) | 24.20 | 5.21 *10^-06^*** |
| Weekend  (6, 32, 49, 278) | 3.57 | 0.35 |
| Week  (6, 13, 93, 253) | 60.38 | 4.70 *10^-14^*** |
| Month  (26, 0, 102, 237) | 102.00 | 3.33 *10^-23^*** |

Note: Each McNemar test was conducted for each response option on number to select that option versus number to not select that option between questions regarding wrist/ankle bands and sticker electrode technology.

*(n = a, b, c, d)* = Frequency of responders to indicate Response Option *X* for questions regarding: *a* = both sticker electrodes and wrist/ankle bands; *b* = sticker electrodes only; *c* = wrist/ankle bands only; *d* = Neither wrist/ankle bands nor sticker electrodes. *Adj. p Value (Bonf. Corr.)* = Bonferroni corrected *p* value. *** denotes significance.

### S5 Table 4: McNemar tests for preferred length of participation between smart suits and wrist/ankle bands

McNemar post hoc tests for Bhapkar test on response frequency for question regarding preferred length of participation between smart suits and wrist/ankle bands.

| Response Option  (n = a, b, c, d) | McNemar *χ^2^* | Adj. p Value  (Bonf. Corr.) |
| --- | --- | --- |
| Not at all  (3, 0, 1, 259) | 1.00 | 1.00 |
| Once  (7, 13, 11, 332) | 0.17 | 1.00 |
| Now and Then  (34, 36, 26, 267) | 1.61 | 1.00 |
| Weekend  (23, 42, 31, 267) | 1.66 | 1.00 |
| Week  (47, 52, 52, 212) | 0.00 | 1.00 |
| Month  (83, 23, 45, 212) | 7.12 | 0.05*** |

Note: Each McNemar test was conducted for each response option on number to select that option versus number to not select that option between questions regarding smart suits and wrist/ankle band technology.

*(n = a, b, c, d)* = Frequency of responders to indicate Response Option *X* for questions regarding: *a* = both smart suits and wrist/ankle bands; *b* = smart suits only; *c* = wrist/ankle band only; *d* = Neither smart suits nor wrist/ankle band. *Adj. p Value (Bonf. Corr.)* = Bonferroni corrected p value. *** denotes significance.

# S6: Additional demographics

| S6 Table 1: Family disorders Parental disorder diagnoses for mother and father of each participating family. | | |
| --- | --- | --- |
|  | % | |
| **Parental Demographic** | Mother | Father |
| Disorder  Autism Spectrum Disorder  Genetic Syndrome  Attention Deficit Disorder  Anxiety Disorder  Depression  Other  None  Prefer not to say  Skipped | 1.20  .70  2.90  12.00  16.80  3.40  53.90  .70  21.22 | 2.20  .50  2.00  3.40  8.50  1.50  55.10  .70  29.26 |

## S6 Table 2: Primary caregiver per day

Percentage of families who have each of the listed family members as the primary caregiver as of the youngest child per day of the week.

|  | % | | | | | | |
| --- | --- | --- | --- | --- | --- | --- | --- |
| Family member | Monday | Tuesday | Wednesday | Thursday | Friday | Saturday | Sunday |
| Mother | 68.8 | 65.9 | 64.4 | 63.4 | 72.7 | 88 | 87.8 |
| Father | 8.8 | 8.8 | 8.3 | 9.8 | 10 | 42.4 | 42.4 |
| Grandparent | 3.4 | 4.9 | 6.1 | 4.9 | 1.2 | .7 | .7 |
| Nanny | 3.7 | 4.1 | 4.4 | 4.1 | 2.9 | 0 | 0 |
| Nursery | 17.6 | 19.3 | 17.1 | 19.8 | 15.6 | 0 | 0 |
| Other | 3.7 | 3.7 | 3.7 | 3.2 | 2.4 | 1 | 1.2 |
| PNA* | .2 | 0 | 0 | 0 | 0 | 0 | 0 |

*Note.* PNA* refers to multiple choice answer of ‘Prefer not to answer’*.* Those that skipped the question and therefore did not contribute an answer (n = 9) have been included in the percentage calculation.

# S7: Table summaries of Post Hoc Analysis for smart suits and stickers

## S7 Table 1: Dun tests for FANOVA on practicality

Dunn post-hocs Test for FANOVA on rating regarding practicality of using smart suits and sticker electrodes for a “Short” or “Extended” period.

| Dunn Test Comparison | |  |  |
| --- | --- | --- | --- |
| Question 1  *(M, SD)* | Question 2  *(M, SD)* | Standardised Test Statistic | Adj. p Value  (Bonf. Corr.) |
| Smart Suits “Short”  (3.50, 0.62) | Smart Suits “Extended”  (2.85, 0.88) | 7.62 | < 0.001*** |
|  | Sticker Electrodes “Short”  (2.14, 0.93) | 15.05 | < 0.001*** |
|  | Sticker Electrodes “Extended”  (1.58, 0.74) | 22.19 | < 0.001*** |
| Smart Suits “Extended”  (2.85, 0.88) | Sticker Electrodes “Short”  (2.14, 0.93) | 7.43 | < 0.001*** |
|  | Sticker Electrodes “Extended”  (1.58, 0.74) | 14.56 | < 0.001*** |
| Sticker Electrodes “Short”  (2.14, 0.93) | Sticker Electrodes “Extended”  (1.58, 0.74) | 7.14 | < 0.001*** |

*Note:* Adj. p Value (Bonf. Corr.) = Bonerfoni corrected p value. *** denotes significance.

## S7 Note A: Comparison of length of participation between smart suits and stickers

The acceptable duration for smart suits was not equally distributed *χ^2^*(5, *N* = 382) = 148.14, *p* < .001, *Cramer’s V* = 0.28); participants were more likely to judge that they would use them for a “Week” or a “Month” than “Day” (S7, Table 2; *p* = 0.05 – *p* < 0.001). Participants also showed duration preferences for sensing electrode stickers, *χ^2^*(5, *N* = 375) = 172.47, *p* < .001, *Cramer’s V* = 0.30) technologies, more frequently opting for “Once” or “Now and Then” over all other options (S7, Table 3; *p* < 0.001). Of the 76% of responders who changed their response between smart suits and sensing electrode stickers, 97% chose longer periods of study duration for smart suits (see S7, Table 4 for McNemar post-hoc test).

### S7 Table 2: Binominal tests for Chi-Square test for length of smart suit participation

Binomial post hoc tests for Chi-Square test on distribution of response frequency for question regarding preferred length of participation for smart suits technology.

| Binomial Comparisons | |  |  |
| --- | --- | --- | --- |
| Response 1  (n) | Response 2  (n) | Adj. p Value  (Bonf. Corr.) | Relative Risk* |
| Not at all  (5) | Once  (21) | 0.04*** | 0.38 |
|  | Now and Then  (74) | 1.20 *10^-15^*** | 0.13 |
|  | Weekend  (65) | 3.32 *10^-13^*** | 0.14 |
|  | Week  (104) | 5.67 *10^-24^*** | 0.09 |
|  | Month  (113) | 1.65 *10^-26^*** | 0.08 |
| Once  (21) | Now and Then  (74) | 6.37 *10^-07^*** | 0.44 |
|  | Weekend  (65) | 3.23 *10^-05^*** | 0.49 |
|  | Week  (104) | 3.14 *10^-13^*** | 0.33 |
|  | Month  (113) | 2.94 *10^-15^*** | 0.31 |
| Now and Then  (74) | Weekend  (65) | 1.00 | 1.06 |
|  | Week  (104) | 0.44 | 0.83 |
|  | Month  (113) | 0.08 | 0.80 |
| Weekend  (65) | Week  (104) | 0.05*** | 0.77 |
|  | Month  (113) | 5.5 *10^-03^*** | 0.73 |
| Week  (104) | Month  (113) | 1.00 | 0.96 |

Note: *Adj. p Value (Bonf. Corr.)* = Bonferroni corrected p value. *Relative risk denotes probability ratio of participant choosing response 1 over response 2. *** denotes significance.

### S7 Table 3: Binominal tests for Chi-Square test for length of sticker electrodes participation

Binomial post hoc tests for Chi-Square test on distribution of response frequency for question regarding preferred length of participation with sticker electrode technology.

| Binomial Comparisons | |  |  |
| --- | --- | --- | --- |
| Response 1  (n) | Response 2  (n) | Adj. p Value  (Bonf. Corr.) | Relative Risk* |
| Not at all  (49) | Once  (121) | 4.88 *10^-07^*** | 0.58 |
|  | Now and Then  (121) | 4.88 *10^-07^*** | 0.58 |
|  | Weekend  (38) | 1.00 | 1.13 |
|  | Week  (19) | 5.37 *10^-03^*** | 1.44 |
|  | Month  (27) | 0.23 | 1.29 |
| Once  (121) | Now and Then  (121) | 1.00 | 1.00 |
|  | Weekend  (38) | 4.12 *10^-10^*** | 1.52 |
|  | Week  (19) | 3.48 *10^-18^*** | 1.73 |
|  | Month  (27) | 3.12 *10^-14^*** | 1.64 |
| Now and Then  (121) | Weekend  (38) | 4.12 *10^-10^*** | 1.52 |
|  | Week  (19) | 3.48 *10^-18^*** | 1.73 |
|  | Month  (27) | 3.12 *10^-14^*** | 1.64 |
| Weekend  (38) | Week  (19) | 0.25 | 1.33 |
|  | Month  (27) | 1.00 | 1.17 |
| Week  (19) | Month  (27) | 1.00 | 0.83 |

Note: *Adj. p Value (Bonf. Corr.)* = Bonferroni corrected p value. *Relative risk denotes probability ratio of participant choosing response 1 over response 2. *** denotes significance.

### S7 Table 4: McNemar tests for Bhapkar test on length of participation between smart suits and sticker electrodes.

McNemar post hoc tests for Bhapkar test on response frequency for question regarding preferred length of participation between smart suits and sticker electrodes.

| Response Option  (n = a, b, c, d) | McNemar *χ^2^* | Adj. p Value  (Bonf. Corr.) |
| --- | --- | --- |
| Not at all  (2, 3, 46, 321) | 37.73 | 4.86 *10^-09^*** |
| Once  (16, 4, 104, 248) | 92.60 | 3.85 *10^-21^*** |
| Now and Then  (28, 42, 93, 209) | 19.27 | 6.82 *10^-05^*** |
| Weekend  (8, 57, 29, 278) | 9.12 | 0.02*** |
| Week  (9, 92, 10, 261) | 65.92 | 2.82 *10^-15^*** |
| Month  (26, 85, 1, 260) | 82.05 | 7.98 *10^-19^*** |

*Note:* Each McNemar test was conducted for each response option on number to select that option versus number to not select that option between questions regarding smart suits and sticker electrode technology.

*(n = a, b, c, d)* = Frequency of responders to indicate Response Option *X* for questions regarding: *a* = both smart suits and sticker electrodes; *b* = smart suits only; *c* = sticker electrodes only; *d* = Neither smart suits nor sticker electrodes. *Adj. p Value (Bonf. Corr.)* = Bonferroni corrected p value. *** denotes significance.

# S8: Table summaries of Post Hoc Analysis for smartphones

## S8 Table 1: Dunn tests for Cochran’s test on preferred response time

Dunn post-hocs Test for Cochran’s test regarding preferred response time.

| Dunn Test Comparison | |  |  |
| --- | --- | --- | --- |
| Question 1  (% indicated Yes) | Question 2  (% indicated Yes) | Standardised Test Statistic | Adj. p Value  (Bonf. Corr.) |
| Anytime  (36.70) | Never  (5.70) | 8.83 | < 0.001*** |
|  | Morning  (14.00) | 6.45 | < 0.001*** |
|  | Afternoon  (11.30) | 7.21 | < 0.001*** |
|  | Evening  (39.40) | -0.76 | 1.00 |
| Evening  (39.40) | Never  (5.70) | 9.59 | < 0.001*** |
|  | Morning  (14.00) | 7.22 | < 0.001*** |
|  | Afternoon  (11.30) | 7.98 | <0.001*** |
| Afternoon  (11.30) | Never  (5.70) | 1.61 | 1.00 |
|  | Morning  (14.00) | -0.76 | 1.00 |
| Morning  (14.00) | Never  (5.70) | -2.78 | 0.18 |

*Note:* Adj. p Value (Bonf. Corr.) = Bonerfoni corrected p value. *** denotes significance.

## S8 Table 2: Binominal tests for Chi-Square test on preferred number of prompts

Binomial post hoc tests for Chi-Square test on distribution of response frequency for question regarding preferred number of prompts to smartphone technology.

| Binomial Comparison | |  |  |
| --- | --- | --- | --- |
| Response 1  (n) | Response 2  (n) | Adj. p Value  (Bonf. Corr.) | Relative Risk* |
| One  (38) | Two  (125) | 7.43 *10^-11^*** | 0.47 |
|  | Four  (119) | 9.60 *10^-10^*** | 0.48 |
|  | Six  (26) | 1.00 | 1.19 |
|  | No Limit  (26) | 0.37 | 1.31 |
|  | None  (20) | 4.68 *10^-05^*** | 1.69 |
| Two  (125) | Four  (119) | 1.00 | 1.02 |
|  | Six  (26) | 1.50 *10^-15^*** | 1.66 |
|  | No Limit  (26) | 1.40 *10^-18^*** | 1.72 |
|  | None  (20) | 6.87 *10^-28^*** | 1.89 |
| Four  (119) | Six  (26) | 3.06 *10^-14^*** | 1.64 |
|  | No Limit  (26) | 3.65 *10^-17^*** | 1.71 |
|  | None  (20) | 3.16 *10^-26^*** | 1.89 |
| Six  (26) | No Limit  (26) | 1.00 | 1.13 |
|  | None  (20) | 0.02*** | 1.58 |
| No Limit  (20) | None  (20) | 0.29 | 1.48 |

Note: *Adj. p Value (Bonf. Corr.)* = Bonferroni corrected p value. *Relative risk denotes probability ratio of participant choosing response 1 over response 2. *** denotes significance

# S9: Table summaries of Post Hoc Analysis for Privacy

## S9 Table 1: Dunn tests for FANOVA test on video versus audio recording likelihood

Dunn post-hoc tests for FANOVA on rating regarding likelihood of using Manually or Automatically processed Body Camera, Cot Camera and Audio recording.

| Dunn Test Comparison | |  |  |
| --- | --- | --- | --- |
| Question 1  *(M, SD)* | Question 2  *(M, SD)* | Standardised Test Statistic | Adj. p Value  (Bonf. Corr.) |
| Body Camera Manual  (3.30) | Body Camera Automatic  (3.39) | -6.93 | < 0.001*** |
|  | Cot Camera Manual  (3.79) | -1.10 | 1.00 |
|  | Cot Camera Automatic  (3.79) | -6.87 | < 0.001*** |
|  | Audio Manual  (3.43) | -1.02 | 1.00 |
|  | Audio Automatic  (3.85) | -7.14 | < 0.001*** |
| Body Camera Automatic  (3.79) | Cot Camera Manual  (3.79) | -5.83 | < 0.001*** |
|  | Cot Camera Automatic  (3.79) | 0.06 | 1.00 |
|  | Audio Manual  (3.43) | 6.00 | < 0.001*** |
|  | Audio Automatic  (3.85) | -0.21 | 1.00 |
| Cot Camera Manual  (3.39) | Cot Camera Automatic  (3.79) | -5.77 | < 0.001*** |
|  | Audio Manual  (3.43) | 0.07 | 1.00 |
|  | Audio Automatic  (3.85) | -6.04 | < 0.001*** |
| Cot Camera Automatic  (3.79) | Audio Manual  (3.43) | 5.84 | < 0.001*** |
|  | Audio Automatic  (3.85) | -0.27 | 1.00 |
| Audio Manual  (3.43) | Audio Automatic  (3.85) | -6.11 | < 0.001*** |

*Note:* Adj. p Value (Bonf. Corr.) = Bonferroni corrected p value. *** denotes significance.

## S9 Table 2: Dunn tests for FANOVA test on video/audio recording practicality

Dunn post-hoc tests for FANOVA on rating regarding practicality of using Manually or Automatically processed Body Camera, Cot Camera and Audio recording for a “Short” or “Extended” period.

| Dunn Test Comparison | |  |  |
| --- | --- | --- | --- |
| Question 1  (*M, SD*) | Question 2  (*M, SD*) | Standardised Test Statistic | Adj. p Value  (Bonf. Corr.) |
| Camera “Short”  (3.11, 0.82) | Camera “Extended”  (2.57, 0.91) | 8.43 | < 0.001*** |
|  | Audio “Short”  (3.11, 0.81) | -0.209 | 1.00 |
|  | Audio “Extended”  (2.63, 0.89) | 7.53 | < 0.001*** |
| Camera “Extended”  (2.57, 0.91) | Audio “Short”  (3.11, 0.81) | -8.64 | < 0.001*** |
|  | Audio “Extended”  (2.63, 0.89) | -0.90 | 1.00 |
| Audio “Short”  (3.11, 0.81) | Audio “Extended”  (2.63, 0.89) | 7.74 | < 0.001*** |

*Note:* Adj. p Value (Bonf. Corr.) = Bonerfoni corrected p value. *** denotes significance.

## S9 Note A. Comparison of preferred length of participation.

Most parents favoured participation time “Now and Then” for all technologies except automatically processed video, for which they indicated they would be happy to use for at least a “Month” (Main text: Fig 6c). Chi-Square testing indicated ratings as unevenly distributed for all four recording device/processing option combinations, with medium effect: Manually processed video, *χ^2^*(5, *N* = 359) = 46.11, *p* < .001, *Cramer’s V* = 0.16; Automatically processed video, *χ^2^*(5, *N* = 361) = 50.31, *p* < .001, *Cramer’s V* = 0.17; Manually processed audio, *χ^2^*(5, *N* = 344) =51.02, *p* < .001, *Cramer’s V* = 0.17; Automatically processed Audio, *χ^2^*(5, *N* = 344) = 44.43, *p* < .001, *Cramer’s V* = 0.16. For automatically processed video recording, “Month” was deemed the most preferred option whereas “Now and Then” was indicated as the most preferred option for all other combinations of recording device/processing option (i.e. manual video, manual audio and automatic audio). Pairwise comparisons with Bonferroni adjusted binomial test partially confirmed this observation as significant (S8 Table 3 - 6).

Participants opted for longer periods of privacy-preserved (i.e. automatically processed) video and audio recording over manual processing. Bhapkar tests revealed automatic processing increased the length of time participants were willing to record with both video, *χ^2^*(5, *N* = 358) = 59.40, *p* < .001, and audio, *χ^2^*(5, *N* = 343) = 56, *p* < .001. For video recording, of the 36% of responders who changed their response between manual and automatic video processing questions, 80% chose longer periods of study duration for privacy-preserving technology such as automatic on-collection processing. McNemar post-hoc tests with Bonferroni adjustments (S8 Table 7) revealed proportions for automatic processing versus manual processing changed significantly for “Now and Then” (*p* < .001), “Weekend” (*p =* 0.02), and “Month” (*p* = 0.02). Of the 54%, 62%, and 38% of responders who changed their response when selecting “Now and Then”, “Weekend” or “Month” for either automatic and manual video processing, respectively, 87%, 80% and 74% chose relatively longer durations of participation for automatic processing. No significant changes across processing types for other choices were reported (*p* = 0.08 - *p* = 0.50). For audio recording, of the 31% of responders who changed their response between manual and automatic audio processing, 88% chose longer periods of study duration for privacy-preserving technology such as automatic on-collection processing. McNemar post-hoc tests with Bonferroni adjustments (S8 Table 8), revealed proportions for automatic processing versus manual processing changed significantly for “Once” (*p* = 0.001), “Weekend” (*p* = 0.02) and “Month” (*p* < .001). Of the 51%, 56%, and 34% of responders who changed their response when selecting “Once”, “Weekend” or “Month” for either automatic or manual audio processing, respectively, 88%, 90% and 96% chose relatively longer durations of participation for automatic processing.

Selection of “Month” over all other participation length options was stronger for video than audio recording for both types of processing method (i.e. manual and automatic). Bhapkar tests indicated that participants preferred longer durations of recording for video than audio recordings for both manual processing, *χ^2^*(5, *N* = 341) = 18.90, *p* < .001, and automatic processing, *χ^2^*(5, *N* = 343) = 27.10, *p* < .001. Of the 46% (manual processing) and 36% (automatic processing) of responders who changed their response between video and audio technology, 62% (manual processing) and 70% (automatic processing) chose longer periods of study duration for video. Pairwise McNemar post-hoc tests with Bonferroni Corrections (S8 Table 9 - 10), revealed proportions for video recording versus audio recording changed significantly only for “Month” for both manual (*p* = 0.01) and automatic (*p* = 0.01) processing. Of the 56% (manual processing) and 40% (automatic processing) of responders who changed their response from “Month” across audio or video, 73% (manual processing) and 74% (automatic processing) of chose longer periods of participation (i.e. “Week”, “Weekend”, “Now and Then”, “Once” and “Not at All”) for video than audio recording.

### S9 Table 3: Binominal tests for Chi-Square test on preferred length of manual processed video recording

Binomial post hoc tests for Chi-Square test on distribution of response frequency for question regarding preferred length of participation with manually processed video recording.

| Binomial Comparison | |  |  |
| --- | --- | --- | --- |
| Response 1  (n) | Response 2  (n) | Adj. p Value  (Bonf. Corr.) | Relative Risk* |
| Not at all  (28) | Once  (60) | 0.01*** | 0.64 |
|  | Now and Then  (98) | 4.03 *10^-09^*** | 0.444 |
|  | Weekend  (49) | 0.33 | 0.73 |
|  | Week  (53) | 0.12 | 0.69 |
|  | Month  (71) | 2.74 *10^-04^*** | 0.57 |
| Once  (60) | Now and Then  (98) | 0.05*** | 0.76 |
|  | Weekend  (49) | 1.00 | 1.10 |
|  | Week  (53) | 1.00 | 1.06 |
|  | Month  (71) | 1.00 | 0.92 |
| Now and Then  (98) | Weekend  (49) | 9.78 *10^-04^*** | 1.33 |
|  | Week  (53) | 4.69 *10^-03^*** | 1.30 |
|  | Month  (71) | 0.68 | 1.16 |
| Weekend  (49) | Week  (53) | 1.00 | 0.96 |
|  | Month  (71) | 0.82 | 0.82 |
| Week  (53) | Month  (71) | 1.00 | 0.85 |

Note: *Adj. p Value (Bonf. Corr.)* = Bonferroni corrected p value. *Relative risk denotes probability ratio of participant choosing response 1 over response 2. *** denotes significance.

### S9 Table 4: Binominal tests for Chi-Square test on preferred length of automatic processed video recording

Binomial post hoc tests for Chi-Square test on distribution of response frequency for question regarding preferred length of participation with automatically processed video recording.

| Binomial Comparison | |  |  |
| --- | --- | --- | --- |
| Response 1  (n) | Response 2  (n) | Adj. p Value  (Bonf. Corr.) | Relative Risk* |
| Not at all  (19) | Once  (45) | 0.02*** | 0.59 |
|  | Now and Then  (70) | 7.44 *10^-07^*** | 0.43 |
|  | Weekend  (72) | 2.95 *10^-07^*** | 0.42 |
|  | Week  (66) | 4.56 *10^-06^*** | 0.45 |
|  | Month  (89) | 7.70 *10^-11^*** | 0.35 |
| Once  (45) | Now and Then  (70) | 0.37 | 0.78 |
|  | Weekend  (72) | 0.24 | 0.77 |
|  | Week  (66) | 0.86 | 0.81 |
|  | Month  (89) | 2.70 *10^-03^*** | 0.67 |
| Now and Then  (70) | Weekend  (72) | 1.00 | 0.99 |
|  | Week  (66) | 1.00 | 1.03 |
|  | Month  (89) | 1.00 | 0.88 |
| Weekend  (72) | Week  (66) | 1.00 | 1.04 |
|  | Month  (89) | 1.00 | 0.89 |
| Week  (66) | Month  (89) | 1.00 | 0.85 |

Note: *Adj. p Value (Bonf. Corr.)* = Bonferroni corrected p value. *Relative risk denotes probability ratio of participant choosing response 1 over response 2. *** denotes significance.

### S9 Table 5: Binominal tests for Chi-Square test on preferred length of manual processed audio recording

Binomial post hoc tests for Chi-Square test on distribution of response frequency for question regarding preferred length of participation with manually processed audio recording.

| Binomial Comparison | |  |  |
| --- | --- | --- | --- |
| Response 1  (n) | Response 2  (n) | Adj. p Value  (Bonf. Corr.) | Relative Risk* |
| Not at all  (29) | Once  (72) | 3.36 *10^-04^*** | 0.57 |
|  | Now and Then  (98) | 8.95 *10^-09^*** | 0.46 |
|  | Weekend  (51) | 0.27 | 0.73 |
|  | Week  (47) | 0.76 | 0.76 |
|  | Month  (47) | 0.76 | 0.76 |
| Once  (72) | Now and Then  (98) | 0.82 | 0.85 |
|  | Weekend  (51) | 1.00 | 1.17 |
|  | Week  (47) | 0.41 | 1.21 |
|  | Month  (47) | 0.41 | 1.21 |
| Now and Then  (98) | Weekend  (51) | 2.19 *10^-03^*** | 1.32 |
|  | Week  (47) | 4.13 *10^-04^*** | 1.35 |
|  | Month  (47) | 4.13 *10^-04^*** | 1.35 |
| Weekend  (51) | Week  (47) | 1.00 | 1.04 |
|  | Month  (47) | 1.00 | 1.04 |
| Week  (47) | Month  (47) | 1.00 | 1 |

Note: *Adj. p Value (Bonf. Corr.)* = Bonferroni corrected p value. *Relative risk denotes probability ratio of participant choosing response 1 over response 2. *** denotes significance.

### S9 Table 6: Binominal tests for Chi-Square test on preferred length of automatic processed audio recording

Binomial post hoc tests for Chi-Square test on distribution of response frequency for question regarding preferred length of participation with automatically processed audio recording.

| Binomial Comparison | |  |  |
| --- | --- | --- | --- |
| Response 1  (n) | Response 2  (n) | Adj. p Value  (Bonf. Corr.) | Relative Risk* |
| Not at all  (21) | Once  (49) | 0.02*** | 0.60 |
|  | Now and Then  (85) | 3.87 *10^-09^*** | 0.40 |
|  | Weekend  (71) | 2.43 *10^-06^*** | 0.46 |
|  | Week  (49) | 0.02*** | 0.60 |
|  | Month  (69) | 5.82 *10^-06^*** | 0.467 |
| Once  (49) | Now and Then  (85) | 0.04*** | 0.73 |
|  | Weekend  (71) | 0.82 | 0.82 |
|  | Week  (49) | 1.00 | 1.00 |
|  | Month  (69) | 1.00 | 0.83 |
| Now and Then  (85) | Weekend  (71) | 1.00 | 1.09 |
|  | Week  (49) | 0.04*** | 1.27 |
|  | Month  (69) | 1.00 | 1.10 |
| Weekend  (71) | Week  (49) | 0.82 | 1.18 |
|  | Month  (69) | 1.00 | 1.01 |
| Week  (49) | Month  (69) | 1.00 | 0.83 |

Note: *Adj. p Value (Bonf. Corr.)* = Bonferroni corrected p value. *Relative risk denotes probability ratio of participant choosing response 1 over response 2. *** denotes significance.

### S9 Table 7: McNemar tests for Bhapkar test on preferred length of manual v automatic processed video recording

McNemar post hoc tests for Bhapkar test on response frequency for question regarding preferred length of participation between manually processed and automatically processed video recording.

| Response Option  (n = a, b, c, d) | McNemar X^2^ | Adj. p Value  (Bonf. Corr.) |
| --- | --- | --- |
| Not at all  (16, 11, 2, 329) | 6.23 | 0.08 |
| Once  (32, 28, 13, 285) | 5.49 | 0.11 |
| Now and Then  (53, 45, 17, 243) | 12.65 | 2.26 *10^-03^*** |
| Weekend  (33, 16, 38, 271) | 8.96 | 0.02*** |
| Week  (35, 18, 30, 275) | 3.00 | 0.50 |
| Month  (61, 10, 28, 259) | 8.53 | 0.02*** |

Note: Each McNemar test was conducted for each response option on number to select that option versus number to not select that option between questions regarding manually processed and automatically processed video recording.

*(n = a, b, c, d)* = Frequency of responders to indicate Response Option *X* for questions regarding: *a* = both manually and automatically processed video; *b* = manually processed only; *c* = automatically processed only; *d* = Neither manually nor automatically processed video. *Adj. p Value (Bonf. Corr.)* = Bonferroni corrected p value. *** denotes significance.

### S9 Table 8: McNemar tests for Bhapkar test on preferred length of manual v automatic processed audio recording

Binomial post hoc tests for Chi-Square test on distribution of response frequency for question regarding preferred length of participation with manually and automatically processed audio recording.

| Response Option  (n = a, b, c, d) | McNemar X^2^ | Adj. p Value  (Bonf. Corr.) |
| --- | --- | --- |
| Not at all  (19, 10, 2, 312) | 5.33 | 0.13 |
| Once  (39, 33, 9, 262) | 13.71 | 1.28 *10^-03^*** |
| Now and Then  (63, 34, 22, 224) | 2.57 | 0.65 |
| Weekend  (37, 14, 34, 258) | 8.33 | 0.02*** |
| Week  (33, 14, 16, 280) | 0.13 | 1.00 |
| Month  (46, 1, 23, 273) | 20.17 | 4.26 *10^-05^*** |

Note: Each McNemar test was conducted for each response option on number to select that option versus number to not select that option between questions regarding manually processed and automatically processed audio recording.

*(n = a, b, c, d)* = Frequency of responders to indicate Response Option *X* for questions regarding: *a* = both manually and automatically processed audio; *b* = manually processed only; *c* = automatically processed only; *d* = Neither manually nor automatically processed video. *Adj. p Value (Bonf. Corr.)* = Bonferroni corrected p value. *** denotes significance.

### S9 Table 9: McNemar tests for Bhapkar test on preferred length of manual processed video v audio recording

McNemar post hoc tests for Bhapkar test on response frequency for question regarding preferred length of participation between manually processed video and audio recording.

| Response Option  (n = a, b, c, d) | McNemar X^2^ | Adj. p Value  (Bonf. Corr.) |
| --- | --- | --- |
| Not at all  (18, 7, 8, 307) | 0.25 | 1.00 |
| Once  (29, 24, 43, 245) | 5.39 | 0.12 |
| Now and Then  (53, 40, 45, 203) | 0.30 | 1.00 |
| Weekend  (22, 27, 28, 264) | 0.02 | 1.00 |
| Week  (27, 26, 20, 268) | 0.79 | 1.00 |
| Month  (35, 33, 12, 261) | 9.80 | 0.01*** |

Note: Each McNemar test was conducted for each response option on number to select that option versus number to not select that option between questions regarding automatically processed video and audio recording.

*(n = a, b, c, d)* = Frequency of responders to indicate Response Option *X* for questions regarding: *a* = both camera and audio recording; *b* = camera only; *c* = audio only; *d* = Neither camera nor audio recording. *Adj. p Value (Bonf. Corr.)* = Bonferroni corrected p value. *** denotes significance.

### S9 Table 10: McNemar tests for Bhapkar test on preferred length of automatic processed video v audio recording

McNemar post hoc tests for Bhapkar test on response frequency for question regarding preferred length of participation between automatically processed video and audio recording.

| Response Option  (n = a, b, c, d) | McNemar X^2^ | Adj. p Value  (Bonf. Corr.) |
| --- | --- | --- |
| Not at all  (13, 4, 7, 319) | 0.82 | 1.00 |
| Once  (24, 14, 25, 280) | 3.10 | 0.47 |
| Now and Then  (48, 20, 37, 238) | 5.07 | 0.15 |
| Weekend  (39, 29, 32, 243) | 0.15 | 1.00 |
| Week  (38, 26, 11, 268) | 6.08 | 0.08 |
| Month  (59, 29, 10, 245) | 9.26 | 0.01*** |

Note: Each McNemar test was conducted for each response option on number to select that option versus number to not select that option between questions regarding automatically processed video and audio recording.

*(n = a, b, c, d)* = Frequency of responders to indicate Response Option *X* for questions regarding: *a* = both camera and audio recording; *b* = camera only; *c* = audio only; *d* = Neither camera nor audio recording. *Adj. p Value (Bonf. Corr.)* = Bonferroni corrected p value. *** denotes significance.

## S9 Note B: Comparison of when willing to participate per day.

Most parents were happy to participate in audio/video recordings during both “Day and Night” (Main text: Fig 6d). Chi-Square testing indicated ratings as unevenly distributed for all four recording device/processing option combinations, with strong effect: Manually processed video, *χ^2^*(4, *N* = 358) = 448.65, *p* < .001, *Cramer’s V* = 0.56; Automatically processed video, *χ^2^*(4, *N* = 353) = 612.80, *p* < .001, *Cramer’s V* = 0.66; Manually processed audio, *χ^2^*(4, *N* = 342) = 397.77, *p* < .001, *Cramer’s V* = 0.54; Automatically processed audio, *χ^2^*(4, *N* = 342) = 555.90, *p* < .001, *Cramer’s V* = 0.64. Pairwise comparisons with Bonferroni corrected binomial tests (S8 Table 11 - 14) confirmed “Day and Night” as significantly most preferred over all other options for all technologies (*p* < 0.001).

Selection of “Day and Night” over “Day”, “Night”, “Never” or “Specific Time” was stronger for automatic vs manual processing. Bhapkar tests revealed processing method (i.e. automatic or manual) cased significant changes to individuals’’ preferred recording time for both video recording, *χ^2^*(4, *N* = 350) = 20.10, *p* < .001, and audio recording, *χ^2^*(4, *N* = 337) = 22.79, *p* < .001. For video recording, of the 15% of responders who changed their response between manual and automatic video processing questions, 75% chose longer period of recording per day for privacy-preserving technology such as automatic on-collection processing. McNemar post-hoc tests with Bonferroni adjustments (S8 Table 15), revealed proportions of responses to length of time choices for automatic processing versus manual processing changed significantly for “Day and Night” (*p* < .001). Of the 15%, of responders who changed their response when selecting “Day and Night” for either automatic or manual audio processing, 66% chose relatively longer period of recording per day when automatic processing is applied. For audio recording, of the 15% of responders who changed their response between manual and automatic audio processing questions, 73% chose longer periods of recording per day for privacy-preserving technology such as automatic on-collection processing. McNemar post-hoc tests with Bonferroni adjustments (S8 Table 16), revealed proportions of responses to length of time choices change for “Never: (*p* < .001) and “Day and Night” (*p* < .001). Of the 34%, and 17% of responders who changed their response when selecting “Never”, or “Day and Night” for either automatic or manual audio processing, respectively, 70% and 81% chose relatively longer periods of recording per day when automatic processing is applied. No other significant changes across processing types for other length of time choices were reported for audio recording (*p* = 0.21 – 1.00).

Participants opted for longer periods of video recording per day over audio recording, only when the recordings were to be manually processed. Bhapkar tests revealed technology (video or audio) caused significant changes to individuals preferred recording time when manually processed, *χ^2^*(4, *N* = 335) = 14.07, *p* < 0.001. Of the 30% of responders who changed their response between manually processed video and audio, 56% chose longer periods of recording per day for video. McNemar post-hoc tests with Bonferroni Corrections (S8 Table 17), revealed proportions for manual video recording versus manual audio recording changed significantly only for “Night” (*p* = 0.01). Of the 82% of responders who changed their response when selecting “Night” for either manual video or audio recording, 47% chose relatively longer periods of recording per day for video. Although 25% of responders changed response between recording device, Bhapkar tests showed no significant effect of technology on individuals’ preferred participation time of automatic recordings, *χ^2^*(4, *N* = 331) = 6.53, *p* = 0.16.

### S9 Table 11: Binomial tests for Chi-Square on preferred time per day of participation with manually processed video recording.

Binomial post hoc tests for Chi-Square test on distribution of response frequency for question regarding preferred time per day of participation with manually processed video recording.

| Binomial Comparison | |  | |
| --- | --- | --- | --- |
| Response 1  (n) | Response 2  (n) | Adj. p Value  (Bonf. Corr.) | Relative Risk* |
| Day and Night  (231) | Day  (43) | 2.74 *10^-31^*** | 1.69 |
|  | Night  (20) | 1.13 *10^-45^*** | 1.84 |
|  | Never  (25) | 5.97 *10^-42^*** | 1.80 |
|  | Specific Time  (39) | 2.31 *10^-33^*** | 1.71 |
| Day  (43) | Night  (20) | 0.05*** | 1.37 |
|  | Never  (25) | 0.38 | 1.26 |
|  | Specific Time  (39) | 1.00 | 1.05 |
| Night  (20) | Never  (25) | 1.00 | 0.89 |
|  | Specific Time  (39) | 0.18 | 0.68 |
| Never  (25) | Specific Time  (39) | 1.00 | 0.78 |

Note: *Adj. p Value (Bonf. Corr.)* = Bonferroni corrected p value. *Relative risk denotes probability ratio of participant choosing response 1 over response 2. *** denotes significance.

### S9 Table 12: Binomial tests for Chi-Square on preferred length per day with automatically processed video recording.

Binomial post hoc tests for Chi-Square test on distribution of response frequency for question regarding preferred when willing to participate per day with automatically processed video recording.

| Binomial Comparisons | |  |  |
| --- | --- | --- | --- |
| Response 1  (n) | Response 2  (n) | Adj. p Value  (Bonf. Corr.) | Relative Risk* |
| Day and Night  (256) | Day  (36) | 4.69 *10^-41^*** | 1.75 |
|  | Night  (16) | 7.69 *10^-56^*** | 1.88 |
|  | Never  (16) | 7.69 *10^-56^*** | 1.88 |
|  | Specific Time  (29) | 1.45 *10^-45^*** | 1.80 |
| Day  (36) | Night  (16) | 0.08 | 1.38 |
|  | Never  (16) | 0.08 | 1.38 |
|  | Specific Time  (29) | 1.00 | 1.12 |
| Night  (16) | Never  (16) | 1.00 | 1.00 |
|  | Specific Time  (29) | 0.72 | 0.71 |
| Never  (16) | Specific Time  (29) | 0.72 | 0.71 |

Note: *Adj. p Value (Bonf. Corr.)* = Bonferroni corrected p value. *Relative risk denotes probability ratio of participant choosing response 1 over response 2. *** denotes significance.

### S9 Table 13: Binomial tests for Chi-Square on preferred length per day with manually processed audio recording.

Binomial post hoc tests for Chi-Square test on distribution of response frequency for question regarding when willing to participate per day with manually processed audio recording.

| Binomial Comparison | |  |  |
| --- | --- | --- | --- |
| Response 1  (n) | Response 2  (n) | Adj. p Value  (Bonf. Corr.) | Relative Risk* |
| Day and Night  (213) | Day  (51) | 1.03 *10^-23^*** | 1.61 |
|  | Night  (7) | 5.51 *10^-53^*** | 1.94 |
|  | Never  (30) | 3.54 *10^-34^*** | 1.75 |
|  | Specific Time  (41) | 3.32 *10^-28^*** | 1.68 |
| Day  (51) | Night  (7) | 2.40 *10^-08^*** | 1.76 |
|  | Never  (30) | 0.26 | 1.26 |
|  | Specific Time  (41) | 1.00 | 1.11 |
| Night  (7) | Never  (30) | 9.20 *10^-03^*** | 0.38 |
|  | Specific Time  (41) | 6.24 *10^-06^*** | 0.29 |
| Never  (30) | Specific Time  (41) | 1.00 | 0.84 |

Note: *Adj. p Value (Bonf. Corr.)* = Bonferroni corrected p value. *Relative risk denotes probability ratio of participant choosing response 1 over response 2. *** denotes significance.

### S9 Table 14: Binomial tests for Chi-Square on preferred length per day with automatically processed audio recording.

Binomial post hoc tests for Chi-Square test on distribution of response frequency for question regarding preferred when willing to participate per day with automatically processed audio recording.

| Binomial Comparison | |  |  |
| --- | --- | --- | --- |
| Response 1  (n) | Response 2  (n) | Adj. p Value  (Bonf. Corr.) | Relative Risk* |
| Day and Night  (241) | Day  (45) | 1.47 *10^-32^*** | 1.69 |
|  | Night  (7) | 4.79 *10^-61^*** | 1.94 |
|  | Never  (19) | 3.76 *10^-49^*** | 1.85 |
|  | Specific Time  (30) | 4.17 *10^-41^*** | 1.78 |
| Day  (45) | Night  (7) | 6.97 *10^-07^*** | 1.73 |
|  | Never  (19) | 0.02*** | 1.42 |
|  | Specific Time  (30) | 1.00 | 1.20 |
| Night  (7) | Never  (19) | 0.29 | 0.54 |
|  | Specific Time  (30) | 1.91 *10^-03^*** | 0.38 |
| Never  (19) | Specific Time  (3) | 1.00 | 0.78 |

Note: *Adj. p Value (Bonf. Corr.)* = Bonferroni corrected p value. *Relative risk denotes probability ratio of participant choosing response 1 over response 2. *** denotes significance.

### S9 Table 15: McNemar tests for Bhapkar test on when willing to participate per day between manually processed and automatically processed video recording.

McNemar post hoc tests for Bhapkar test on response frequency for question regarding preferred when willing to participate per day between manually processed and automatically processed video recording.

| Response Option  (n = a, b, c, d) | McNemar X^2^ | | Adj. p Value  (Bonf. Corr.) | |  |
| --- | --- | --- | --- | --- | --- |
| Day and Night  (221, 8, 33, 88) | | 15.24 | | 4.72 *10^-04^*** | |
| Day  (30, 13, 6, 301) | | 2.58 | | 0.54 | |
| Night  (11, 9, 5, 325) | | 1.14 | | 1.00 | |
| Never  (14, 6, 1, 329) | | 3.57 | | 0.29 | |
| Specific  (23, 15, 6, 306) | | 3.86 | | 0.25 | |

Note: Each McNemar test was conducted for each response option on number to select that option versus number to not select that option between questions regarding manually processed and automatically processed video recording.

*(n = a, b, c, d)* = Frequency of responders to indicate Response Option *X* for questions regarding: *a* = both manually and automatically processed video; *b* = manually processed only; *c* = automatically processed only; *d* = Neither manually nor automatically processed video. *Adj. p Value (Bonf. Corr.)* = Bonferroni corrected p value. *** denotes significance.

### S9 Table 16: McNemar tests for Bhapkar test on when willing to participate per day between manually processed and automatically processed audio recording.

McNemar post hoc tests for Bhapkar test on response frequency for question regarding preferred time of participation between manually processed and automatically processed audio recording.

| Response Option  (n = a, b, c, d) | McNemar X^2^ | Adj. p Value  (Bonf. Corr.) |
| --- | --- | --- |
| Day and Night  (204, 34, 8, 91) | 16.10 | 3.01 *10^-04^*** |
| Day  (35, 9, 15, 278) | 1.50 | 1.00 |
| Night  (5, 2, 2, 328) | 0.00 | 1.00 |
| Never  (19, 0, 10, 308) | 10.00 | 7.83 *10^-03^*** |
| Specific  (22, 7, 17, 291) | 4.17 | 0.21 |

Note: Each McNemar test was conducted for each response option on number to select that option versus number to not select that option between questions regarding manually processed and automatically processed audio recording.

*(n = a, b, c, d)* = Frequency of responders to indicate Response Option *X* for questions regarding: *a* = both manually and automatically processed audio; *b* = manually processed only; *c* = automatically processed only; *d* = Neither manually nor automatically processed video. *Adj. p Value (Bonf. Corr.)* = Bonferroni corrected p value. *** denotes significance.

### S9 Table 17: McNemar tests for Bhapkar test on when willing to participate per between manually processed video and audio recording.

McNemar post hoc tests for Bhapkar test on response frequency for question regarding preferred time of participation between manually processed video and audio recording.

| Response Option  (n = a, b, c, d) | | McNemar X^2^ | | Adj. p Value  (Bonf. Corr.) | |
| --- | --- | --- | --- | --- | --- |
| Day and Night  (176, 42, 32, 85) | 1.35 | | 1.00 | |  |
| Day  (17, 23, 33, 262) | 1.79 | | 0.91 | |  |
| Night  (4, 16, 3, 312) | 8.89 | | 0.01*** | |  |
| Never  (15, 7, 14, 299) | 2.33 | | 0.63 | |  |
| Specific  (21, 14, 20, 280) | 1.06 | | 1.00 | |  |

Note: Each McNemar test was conducted for each response option on number to select that option versus number to not select that option between questions regarding automatically processed video and audio recording.

*(n = a, b, c, d)* = Frequency of responders to indicate Response Option *X* for questions regarding: *a* = both camera and audio recording; *b* = camera only; *c* = audio only; *d* = Neither camera nor audio recording. *Adj. p Value (Bonf. Corr.)* = Bonferroni corrected p value. *** denotes significance.

# S10: Table summaries of Post Hoc Analysis for Data Sharing

## S10 Table 1: Binomial tests for Chi-Square on preferred data sharing for anonymous data.

Binomial post hoc tests for Chi-Square test on distribution of response frequency for question regarding preferred data sharing for anonymous data.

| Binominal Comparison | |  |  |
| --- | --- | --- | --- |
| Response 1  (n) | Response 2  (n) | Adj. p Value  (Bonf. Corr.) | Relative Risk* |
| Baby Lab  (35) | UK  (17) | 0.10 | 1.35 |
|  | Europe  (13) | 0.01*** | 1.46 |
|  | International  (259) | 1.26 *10^-42^*** | 0.24 |
| UK  (17) | Europe  (13) | 1.00 | 1.13 |
|  | International  (259) | 5.63 *10^-56^*** | 0.12 |
| Europe  (13) | International  (259) | 8.91 *10^-60^*** | 0.10 |

Note: *Adj. p Value (Bonf. Corr.)* = Bonferroni corrected p value. *Relative risk denotes probability ratio of participant choosing response 1 over response 2. *** denotes significance.

## S10 Table 2: Binomial tests for Chi-Square on preferred data sharing for non-anonymous data.

Binomial post hoc tests for Chi-Square test on distribution of response frequency for question regarding preferred data sharing for non-anonymous data.

| Binomial Comparison | |  |  |
| --- | --- | --- | --- |
| Response 1  (n) | Response 2  (n) | Adj. p Value  (Bonf. Corr.) | Relative Risk* |
| Baby Lab  (151) | UK  (33) | 3.09 *10^-18^*** | 1.64 |
|  | Europe  (7) | 2.44 *10^-35^*** | 1.91 |
|  | International  (127) | 1.00 | 1.09 |
|  | None  (9) | 2.19 *10^-33^*** | 1.89 |
| UK  (33) | Europe  (7) | 4.23 *10^-04^*** | 1.65 |
|  | All  (127) | 3.31 *10^-13^*** | 0.41 |
|  | None  (9) | 3.00 *10^-3^*** | 1.57 |
| Europe  (7) | International  (127) | 1.27 *10^-28^*** | 0.10 |
|  | None  (9) | 1.00 | 0.88 |
| International  (127) | None  (9) | 8.26 *10^-27^*** | 1.87 |

Note: *Adj. p Value (Bonf. Corr.)* = Bonferroni corrected p value. *Relative risk denotes probability ratio of participant choosing response 1 over response 2. *** denotes significance.

## S10 Table 3: McNemar tests for Bhapkar on preferred data sharing between anonymous and non-anonymous.

McNemar post hoc tests for Bhapkar test on response frequency changes for question regarding preferred data sharing between anonymous and non-anonymous.

| Response Option  (n = a, b, c, d) | McNemar X^2^ | Adj. p Value  (Bonf. Corr.) |
| --- | --- | --- |
| BabyLab  (32, 1, 117, 169) | 114.03 | 6.40 *10^-26^*** |
| UK  (13, 4, 17, 285) | 8.05 | 0.02*** |
| Europe  (3, 10, 4, 302) | 2.57 | 0.54 |
| International  (124, 132, 0, 63) | 132.00 | 7.48 *10^-30^*** |
| None  (0, 0, 9, 310) | 9.00 | 0.01*** |

Note: Each McNemar test was conducted for each response option on number to select that option versus number to not select that option between questions regarding automatically processed video and audio recording.

*(n = a, b, c, d)* = Frequency of responders to indicate Response Option *X* for questions regarding: *a* = both anonymous and non-anonymous data; *b* = anonymous data only; *c* = non-anonymous data only; *d* = Neither anonymous nor non-anonymous data. *Adj. p Value (Bonf. Corr.)* = Bonferroni corrected p value. *** denotes significance.

# S11: Table summaries of Post Hoc Analysis for Data access

## S11 Table 1: Dunn tests for Cochran’s test on viewing each type of data.

Dunn post-hocs Test for Cochran’s test regarding most preferred data to view.

| Dunn Test Comparison | |  |  |
| --- | --- | --- | --- |
| Question 1  (% indicated Yes) | Question 2  (% indicated Yes) | Standardised Test Statistic | Adj. p Value  (Bonf. Corr.) |
| Video  (84.90) | Audio  (77.00) | 2.71 | 1.00 |
|  | Heart Rate  (65.00) | -6.88 | < 0.001*** |
|  | Movement  (68.60) | 5.63 | < 0.001*** |
|  | Sweat  (50.20) | 11.99 | < 0.001*** |
|  | Sleep  (90.9) | -2.09 | 0.56 |
| Audio  (77.00) | Heart Rate  (65.00) | -4.17 | < 0.001*** |
|  | Movement  (68.60) | 2.92 | 0.05*** |
|  | Sweat  (50.20) | -9.28 | <0.001*** |
|  | Sleep  (90.9) | -4.80 | < 0.001*** |
| Heart Rate  (65.00) | Movement  (68.60) | -1.25 | 1.00 |
|  | Sweat  (50.20) | 5.11 | < 0.001*** |
|  | Sleep  (90.9) | -8.97 | < 0.001*** |
| Movement  (68.60) | Sweat  (50.20) | -6.36 | < 0.001*** |
|  | Sleep  (90.9) | 7.71 | < 0.001*** |
| Sweat  (50.20) | Sleep  (90.9) | -14.08 | < 0.001*** |

*Note:* Adj. p Value (Bonf. Corr.) = Bonerfoni corrected p value. *** denotes significance.

## S11 Table 2: Dunn tests for Cochran’s test on ok/deleting each type of data.

Dunn post-hocs Test for Cochran’s test regarding Ok/Delete each type of data.

| Dunn Test Comparison | |  |  |
| --- | --- | --- | --- |
| Question 1  (% indicated Yes) | Question 2  (% indicated Yes) | Standardised Test Statistic | Adj. p Value  (Bonf. Corr.) |
| Video  (80.70) | Audio  (75.50) | 0.144 | 1.00 |
|  | Heart Rate  (15.40) | -18.57 | < 0.001*** |
|  | Movement  (17.50) | -17.97 | < 0.001*** |
|  | Sweat  (13.60) | -19.08 | < 0.001*** |
|  | Sleep  (19.60) | -17.36 | < 0.001*** |
|  | None  (13.40) | -18.22 | < 0.001*** |
| Audio  (75.50) | Heart Rate  (15.40) | -17.12 | < 0.001*** |
|  | Movement  (17.50) | -16.50 | < 0.001*** |
|  | Sweat  (13.60) | -17.62 | <0.001*** |
|  | Sleep  (19.60) | -15.90 | < 0.001*** |
|  | None  (13.40) | -16.76 | < 0.001*** |
| Heart Rate  (15.40) | Movement  (17.50) | -0.60 | 1.00 |
|  | Sweat  (13.60) | 0.52 | 1.00 |
|  | Sleep  (19.60) | -1.20 | 1.00 |
|  | None  (13.40) | 0.34 | 1.00 |
| Movement  (17.50) | Sweat  (13.60) | 1.11 | 1.00 |
|  | Sleep  (19.60) | 0.60 | 1.00 |
|  | None  (13.40) | -0.26 | 1.00 |
| Sweat  (13.60) | Sleep  (19.60) | 1.72 | 1.00 |
|  | None  (13.40) | 0.86 | 1.00 |
| Sleep  (19.60) | None  (13.40) | -0.86 | 1.00 |

*Note:* Adj. p Value (Bonf. Corr.) = Bonerfoni corrected p value. *** denotes significance.

# S12: Table summaries of Post Hoc Analysis for Future Participation

## S12 Table 1: Dunn Test for Chi-Square on interest for future participation

Dunn post-hocs Test for Chi Square test on distribution of response frequency regarding interest in future participation.

| Binominal Comparison | |  |  |
| --- | --- | --- | --- |
| Response 1  (n) | Response 2  (n) | Adj. p Value  (Bonf. Corr.) | Relative Risk* |
| Extremely  (76) | Very  (120) | 0.02*** | 0.78 |
|  | Moderately  (75) | 1.00 | 1.02 |
|  | Slightly  (25) | 3.72 *10^-06^*** | 1.50 |
|  | Not at all  (34) | 7.69 *10^-04^*** | 1.38 |
| Very  (120) | Moderately  (75) | 0.02*** | 1.23 |
|  | Slightly  (25) | 4.37 *10^-15^*** | 1.66 |
|  | Not at all  (34) | 1.90 *10^-11^*** | 1.56 |
| Moderately  (75) | Slightly  (25) | 5.64 *10^-06^*** | 1.50 |
|  | Not at all  (34) | 1.07 *10^-03^*** | 1.38 |
| Slightly  (25) | Not at all  (34) | 1.00 | 0.85 |

*Note:* Adj. p Value (Bonf. Corr.) = Bonerfoni corrected p value. *** denotes significance.

## S12 Table 2: Dun Test for Chi-Square on interest for future participation

Dunn post-hocs Test for Chi Square test on distribution of response frequency regarding wanting to participate in future participation.

| Binomial Comparison | |  |  |
| --- | --- | --- | --- |
| Response 1  (n) | Response 2  (n) | Adj. p Value  (Bonf. Corr.) | Relative Risk* |
| N/A  (128) | Yes  (192) | 1.24 *10^-03^*** | 0.80 |
|  | No  (13) | 1.89 *10^-24^*** | 1.82 |
| Yes  (192) | No  (13) | 1.54 *10^-41^*** | 1.87 |

*Note:* Adj. p Value (Bonf. Corr.) = Bonerfoni corrected p value. *** denotes significance.

## S12 Note A: Reuse of smart suits Analysis.

Response from question as to whether they would allow their child to wear a smart suit that other families had previously used are depicted in Fig 4. Pearson’s Chi Square test revealed a significant difference between the number of responders to indicate “Yes” compared to “No”, *χ^2^*(1, *N* = 377) =102.94, *p* < .001. Cramer’s V revealed large effect size of 0.52.

Fig 4. Response percentage to question on whether they would allow their infant to use suits other families have used.

# S13: Low SES Subset Analysis

Sub-analysis was conducted on 20 responders considered lower SES (lower third annual income and parental education to be no higher than secondary school). Of this subsample, the median income bracket was £20,000 - £29,999 and median material and paternal education was secondary school.

## **Practicability**

**Comparison of likelihood.** Overall, median likelihood responses were higher for smart suits (*Mdn* = 5.00, *M* = 4.60, *SD* = 0.82) than sticker electrodes (*Mdn* = 4.00, *M* = 3,74, *SD* = 1.41), S13 Fig 1a. Wilcox Matched-Pairs Signed-Ranks Test (WMPSR) revealed significantly more participants (n = 10) rated using smart suits more likely than sticker electrodes compared to those who rated smart suits less likely (n = 0), *z* = -2.85, *p* = 0.004, with moderate effect size (*r* = -0.65). Nine people rated participation with each technology as equally likely.

**Comparison of practicality.** Parents from low SES thought using smart suits was more practical over sticker electrodes (S13 Fig 1b). A Friedman’s Analysis of Variance (FANOVA) identified significant differences in rating of practicality between using smart suits for a “Short Period” (*Mdn* = 4.00, *M* = 3.55, *SD* = 0.69), smart suits for an “Extended Period” (*Mdn* = 3.00, *M* = 3.16, *SD* = 0.90), sticker electrodes for a “Short Period” (*Mdn* = 3.00, *M* = 2.53, *SD* = 1.02) and sticker electrodes for an Extended Period” (*Mdn* = 2.00, *M* = 2.26, *SD* = 1.05), *χ^2^*(3, N = 18) = 21.68, p < 0.001. Further analysis indicated a Kendall W of 0.402, indicating an average level of agreement among responders(7). Dunn post-hoc tests with Bonferroni adjustments (S13 Table 1) revealed Smart suits for a “Short Period “was considered significantly more practical: than sticker electrodes for a “Short Period” (*p* = 0.04); Sticker electrodes “Extended period” (*p* = 0.003). Smart suits “Extended Period” was considered more practical than sticker electrodes “Extended period” (*p* = 0.04).

## **Study interaction**

**Comparison of likelihood.** Parents of relatively lower SES compared to the entire sample thought that smartphone usage to be highly likely, S13 Fig 1c. A one-sample Wilcoxon signed-rank test indicated that the likelihood score for using smartphones to interact with the study (*Mdn* = 5.00, *M* = 4.53, *SD* = 1.02) was significantly different from 2.5, Z(N=19) = 24.58, p < .001, with a moderate effect size (r = 5.63).

**Comparison of practicality.** Overall, practicality responses were approximately equal for the using the smartphones for a “Short Period” (*Mdn* = 4.00, *M* = 3.74, *SD* = 0.75) and for an “extended period” (*Mdn* = 4.00, *M* = 3.68, *SD* = 0.75), S13 Fig 1d. A WMPSR was found to not be significant, *z* = -0.58, *p* = 0.56. Two people rated using smartphones for a “Short Period” as more practical, whereas one person rated over an “Extended Period” as more practical. The majority of individuals (n=16) rated each length of time as equally practical.

## **Privacy**

**Comparison of likelihood.** Trends indicated automatic processing of each recording technology to be indicated as slightly more likely to be used than manually processed, though not to a significant degree, S13 Fig 1e. A FANOVA identified an overall non-significant differences in rating of likelihood between manually processed body camera (*Mdn* = 4.00, *M* = 3.79, *SD* = 1.32), automatically processed body camera (*Mdn* = 4.00, *M* = 4.05, *SD* = 1.08), manually processed cot camera (*Mdn* = 4.00, *M* = 4.17, *SD* = 1.10), automatically processed cot camera (*Mdn* = 4.00, *M* = 4.00, *SD* = 1.12), Manually processed audio (*Mdn* = 4.00, *M* = 4.00, *SD* = 1.09) and automatically processed audio (*Mdn* = 4.00, *M* = 4.05, *SD* = 1.13), *χ^2^*(5, n = 18) = 4.18, *p* = 0.52. Further analysis indicated a Kendall W of 0.05, indicating a weak level of agreement among responders.

**Comparison of practicality.** Parents indicated smart suits to be indicated as more practical than sticker electrodes, S13 Fig 1f. A FANOVA identified no significant differences in rating of practicality between participating in the video recording for a “Short Period” (*Mdn* = 3.00, *M* = 3.22, *SD* = 0.55), video recording for an “Extended Period” (*Mdn* = 3.00, *M* = 3.00, *SD* = 0.89), audio recording for a “Short Period” (*Mdn* = 3.00, *M* = 3.16, *SD* = 0.69) and audio recording for an “Extended Period” (*Mdn* = 3.00, *M* = 2.95, *SD* = 0.78), *χ^2^*(3, *N* = 18) = 0.61, *p* < 0.89. Further analysis indicated a Kendall W of 0.01, indicating a weak level of agreement among responders(7).

## **Data sharing**

Trends indicated parents of relatively lower SES were happier to share anonymous data more internationally than non-anonymous data, though this was not to a significant effect (S13 Fig 1g). Bhapkar test indicated no significant effect of technology on individual’s choice to data sharing preference, *χ^2^*(3, *N* = 17) = 6.31, *p* = 0 .09.


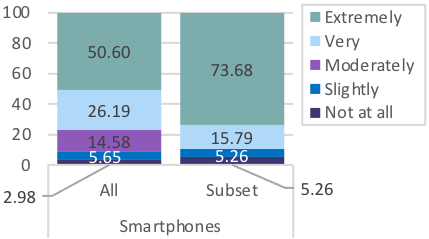


D.

%

%


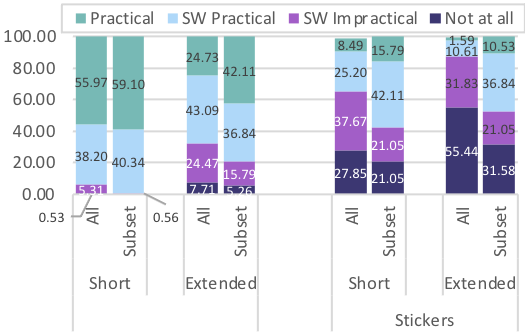


B.


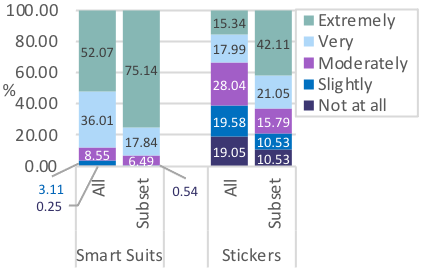


C..

%

E.

%


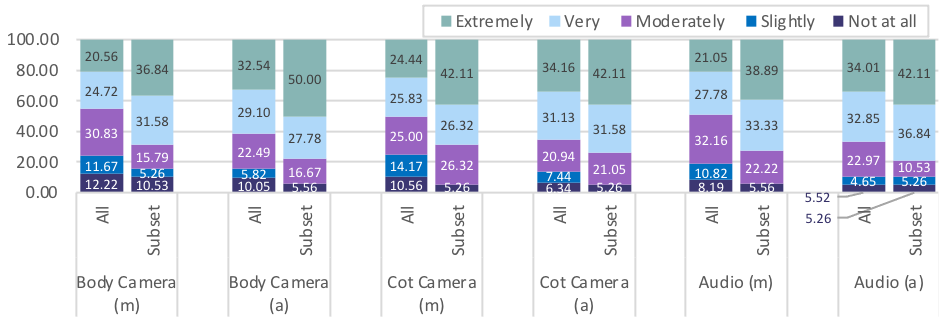

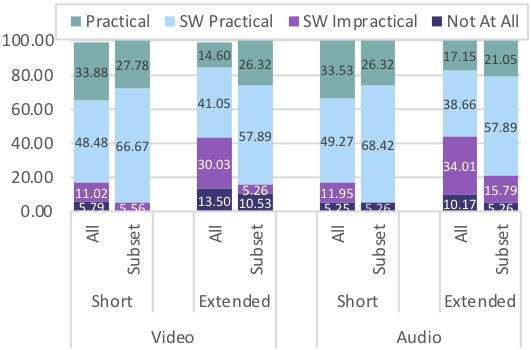


F.


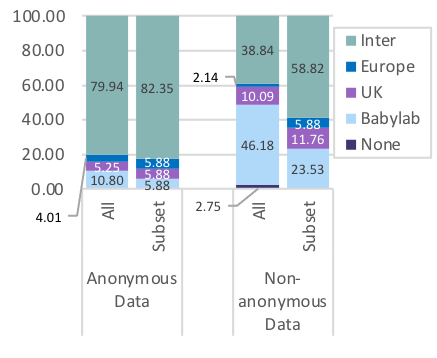


G.

%

%

*S12 Fig 1*. Comparisons of response percentages from low SES subset with entire sample for: *A.* Likelihood of smart suits and sticker electrodes *B.* Practicality of smart suits and sticker electrodes for a “Short Period” or over an “Extended Period”. *C.* Likelihood and D. Practicality of using smartphone to interact with study. *E*. Likelihood of using Camera and Audio technologies if manually process (m) or automatically processed (a). *F.* Practicality of using cameras and audio for a “Short Period” or over an “Extended Period”. *G.* Sharing preference for anonymous and non-anonymous data.

*Note.* Percentages were calculated out of all responders who answered per question, including ‘Prefer not to answer’ responses.


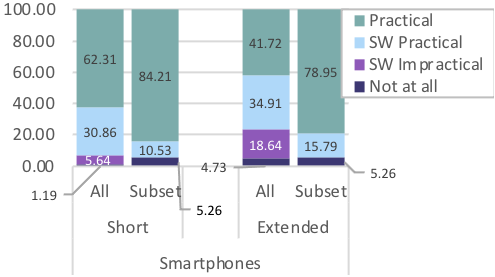


**Feedback**

Similar to the entire sample, parents for relatively lower SES indicated a preference to view sleep, video and audio data, and the ok/delete video and audio data (S13 Fig 2). Cochran's Q test indicated significant differences to the proportions of those who opted a preference to access each type of data to: a) view, χ^2^(5, N = 18) = 22.80, p < .001, with a moderate chance corrected effect size (R = 0.47) ; and b) ok/delete, χ^2^(6, N = 18) = 40.82, p < .001, with a small chance corrected effect size (R = -0.16). For answers regarding access to view data, pairwise post-hoc Dunn tests with Bonferroni adjustments (S13 Table 2) demonstrated sweat data (27.80%) to be significantly less favourable compared with video data (83.30%; *p* = 0.001) and sleep data (88.90%; *p* = 0.001). For the question regarding access to ok/delete data, pairwise post-hoc Dunn tests with Bonferroni adjustments (S13 Table 3) demonstrated video (77.8%) and audio data (77.88%) to be significantly more favourable compared with all other choices (*p* = 0.002 - *p* < 0.001), and no significant differences indicated between these two technologies (*p* = 1.00).


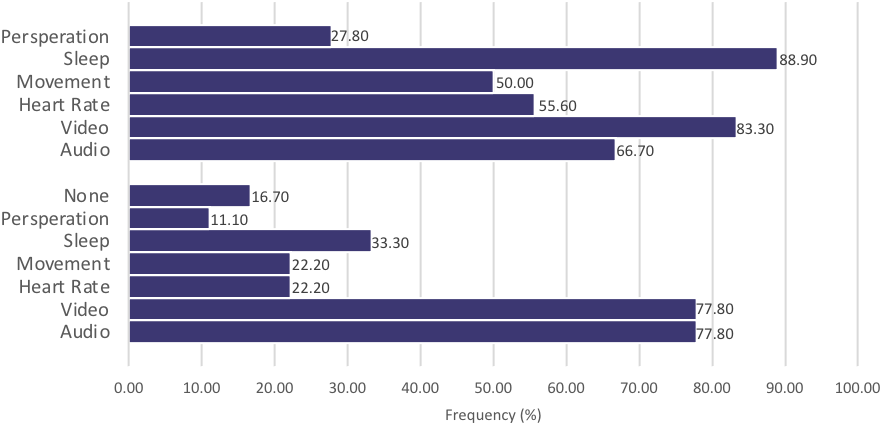


**Access to view**

**Access to OK/Delete**

*S12 Fig* 2. Low SES Percentages of responses for questions concerning accessing different data to view and OK/Delete.

## S13: Table summaries of Post Hoc Analysis of Subset Analysis

### S13 Table 1: Dunn tests FANOVA practicality smart suits and sticker electrodes.

Dunn post-hocs Test for FANOVA on rating regarding practicality of using smart suits and sticker electrodes for a “Short Period” or for an “Extended Period”.

| Dunn Test Comparison | |  |  |
| --- | --- | --- | --- |
| Question 1  *(M, SD)* | Question 2  *(M, SD)* | Standardised Test Statistic | Adj. p Value  (Bonf. Corr.) |
| Smart Suits “Short”  (3.55, 0.69) | Smart Suits “Extended”  (3.16, 0.90) | 0.78 | 1.00 |
|  | Sticker Electrodes “Short”  (2.53, 1.02) | 2.71 | 0.04*** |
|  | Sticker Electrodes “Extended”  (2.26, 1.05) | 3.49 | 0.003*** |
| Smart Suits “Extended”  (3.16, 0.90) | Sticker Electrodes “Short”  (2.53, 1.02) | 1.94 | 0.32 |
|  | Sticker Electrodes “Extended”  (2.26, 1.05) | 2.71 | 0.04*** |
| Sticker Electrodes “Short”  (2.53, 1.02) | Sticker Electrodes “Extended”  (2.26, 1.05) | 0.78 | 1.00 |

*Note:* Adj. p Value (Bonf. Corr.) = Bonerfoni corrected p value. *** denotes significance.

### S13 Table 2: Dunn tests Cochran’s test for viewing each type of data.

Dunn post-hocs Test for Cochran’s test regarding viewing each type of data.

| Dunn Test Comparison | |  |  |
| --- | --- | --- | --- |
| Question 1  (% indicated Yes) | Question 2  (% indicated Yes) | Standardised Test Statistic | Adj. p Value  (Bonf. Corr.) |
| Video  (83.3) | Audio  (66.70) | -1.11 | 1.00 |
|  | Heart Rate  (55.60) | 1.86 | 0.95 |
|  | Movement  (50) | -2.23 | 0.40 |
|  | Sweat  (27.80) | -3.71 | 0.003*** |
|  | Sleep  (88.90) | 0.37 | 1.00 |
| Audio  (66.70) | Heart Rate  (55.60) | 0.74 | 1.00 |
|  | Movement  (50) | -1.11 | 1.00 |
|  | Sweat  (27.80) | 2.60 | 0.14 |
|  | Sleep  (88.90) | 1.48 | 1.00 |
| Heart Rate  (55.60) | Movement  (50) | -0.37 | 1.00 |
|  | Sweat  (27.80) | -1.86 | 0.95 |
|  | Sleep  (88.90) | -2.22 | 0.39 |
| Movement  (50) | Sweat  (27.80) | 1.48 | 1.00 |
|  | Sleep  (88.90) | -2.60 | 0.14 |
| Sweat  (27.80) | Sleep  (88.90) | 4.08 | 0.001*** |

*Note:* Adj. p Value (Bonf. Corr.) = Bonerfoni corrected p value. *** denotes significance.

### S13 Table 3: Dunn tests Cochran’s test for OK/Delete each type of data.

Dunn post-hocs Test for Cochran’s test regarding Ok/Delete each type of data.

| Dunn Test Comparison | |  |  |
| --- | --- | --- | --- |
| Question 1  (% indicated Yes) | Question 2  (% indicated Yes) | Standardised Test Statistic | Adj. p Value  (Bonf. Corr.) |
| Video  (77.80) | Audio  (77.80) | 0.00 | 1.00 |
|  | Heart Rate  (22.20) | -3.60 | 0.007*** |
|  | Movement  (22.20) | -3.60 | 0.007*** |
|  | Sweat  (11.10) | -4.32 | < 0.001*** |
|  | Sleep  (33.30) | -2.88 | 0.08*** |
|  | None  (16.70) | -3.96 | 0.002*** |
| Audio  (77.80) | Heart Rate  (22.20) | -3.60 | 0.007*** |
|  | Movement  (22.20) | -3.60 | 0.007*** |
|  | Sweat  (11.10) | -4.32 | <0.001*** |
|  | Sleep  (33.30) | -3.60 | 0.007*** |
|  | None  (16.70) | -3.96 | 0.002*** |
| Heart Rate  (22.20) | Movement  (22.20) | 0.00 | 1.00 |
|  | Sweat  (11.10) | 0.72 | 1.00 |
|  | Sleep  (33.30) | -0.72 | 1.00 |
|  | None  (16.70) | -0.36 | 1.00 |
| Movement  (22.20) | Sweat  (11.10) | 0.72 | 1.00 |
|  | Sleep  (33.30) | 0.72 | 1.00 |
|  | None  (16.70) | -0.36 | 1.00 |
| Sweat  (11.10) | Sleep  (33.30) | 1.44 | 1.00 |
|  | None  (16.70) | -1.08 | 1.00 |
| Sleep  (33.30) | None  (16.70) | 0.36 | 1.00 |

*Note:* Adj. p Value (Bonf. Corr.) = Bonerfoni corrected p value. *** denotes significance.

# S14 Note A

We explored potential subgroups within our dataset that had differing opinions on with whom they would be happy to share non-anonymous data (i.e. video and audio recordings). To do so, we first selected only those participants who either chose “Babylab” or “International” as their preferred research group to share their non-anonymous data. We conducted a series of Pearson’s chi-square test of independence to determine if there were associations between most preferred research group (“Babylab” or “International”) and the following demographics: child sex, child ethnicity, household annual income and the responder’s relationship to the child. As the majority of the responders were the mother of the subject child, we also tested whether mothers’ education, maternal anxiety (diagnosis/no diagnosis), maternal depression (diagnosis/no diagnosis) associated with preferred choice of data sharing. Maternal education was found to have a significant association with preferred research group to share non-anonymous data (X^2^(1) = 11.86, *p <* 0.001). All other demographics were not-significant (p>0.05). Demographics of child and of parent as per preferred choice of research group to share non-anonymous data are displayed in S13 Fig 1 and S13 Fig 2, respectively.

|  | Babylab | International |
| --- | --- | --- |
| Child’s Sex |  |  |
| Child’s Ethnicity |  |  |
| Responders Relationship to Child |  |  |
| Household Annual Income |  |  |

S14 Fig 1. Child and family demographic break down of those who responded either “Babylab” or “international” as their preferred choice of researcher team with whom to share their non-anonymous data.

|  | Babylab | International |
| --- | --- | --- |
| Maternal Education |  |  |
| Maternal Anxiety |  |  |
| Maternal Depression |  |  |

S14 Fig 2. Maternal demographic breakdown of those who responded either “Babylab” or “international” as their preferred choice of researcher team with whom to share their non-anonymous data.
