# Supplementary material for: A survey on the attitudes of parents with young children on in-home monitoring technologies and study designs for infant research
Source: PLoS One. 2021 Feb 5;16(2):e0245793. doi: 10.1371/journal.pone.0245793 (PMC7864397; doi:10.1371/journal.pone.0245793)
Supplement: Attachment — Submitted filename: Response to Reviewers.docx [file pone.0245793.s004.docx]

Dear Barbara Schouten,

Thank you very much for the consideration of our manuscript for your journal, and thank you to the reviewers who have provided us with comments. We have acknowledged all reviewer comments, and have made the appropriate amendments to the manuscript. We hope the newly revised manuscript now meets PLOS ONE’s standards. Below is a list of reviewer comments (in bold) and our response to each in turn.

**Reviewer #1:**

**The structure of the paper is much more readable, and the authors have been very responsive to prior critiques. I still feel that some of the conclusions are a little broad given the limitations of the survey. I appreciate the need to give specific examples, but I still feel that this may skew the responding in ways that are not known. They have added some wording to acknowledge this, however, and the findings more generally would be of interest to a broader audience even if care must be taken in generalizing these findings to other labs.**

Response: We have added to our conclusions “Although generalisability should be considered,” to re-highlight this limitation (line 1146)

**Reviewer #2:**

**I appreciate the author’s detailed responses to the comments. I think this revision is an improvement, with clearer goals and in a more concise and organized format. I believe that this will be an interesting contribution to the literature. Many of my comments have been addressed appropriately. I have a few more comments below:

Comment 1: Intro: Unclear how smartphones are helping the contextualization? The explanation here doesn’t address contextualization in the ways addressed earlier in the paragraph.**
Response: We have added: “Contextualising information could be collected using smartphones via self-reporting (37). For example, a caregiver could input infant’s current activity in response to a prompt.” (lines 136 – 138)

**Comment 2: Methods: Lines 254 – 259 seem like a list? I think this would be better set up as an enumerated list or with a colon or semi-colons to group these together.**
Response: we have added colon and semi-colon to group this together.

**Comment 3: Results: It seems like there are results presented here that don’t follow from the research questions? For example, the authors pose research questions as related to 1) preferences of lower active vs. higher active technologies, 2) static vs. all-encompassing technologies, and 3) smartphones, but the research questions are framed as preferences and without the different analytic headers as seen in the results re likelihood practicality, and duration with different technologies (although I see the Results and Discussion headers follow consistently).**
Response: We have added subheadings such as “Sticker sensing electrodes versus smart suits” into both the study goals section and the methods that are consistent with the results and discussion to make this clearer. We’ve also reworded some of the method section under these subheadings to make it clearer which questions (e.g. likelihood, practicality and length of participation) were asked in each section.

**Comment 3: Additionally, in the research question the authors mention write/ankle bands, but in the Results we are routed to the supplemental material – making that consistent from the beginning would be helpful (I may be misunderstanding this?). They also use the term sensing bands here in the header – which seems to encompass wrist/ankle bands? The organization in the Results in relation to the Research Questions was still a bit confusing for me.**
Response: We wanted to point the reader to the SM for this analysis as the paper is already very long as previous comments on this paper have pointed out. Also the question/findings from this analysis are similar to the stickers v smart suits so they are more complementary, so they were put in the SM. To make this clearer we’ve removed “sensing bands” from the subheadings and research question, and have “Similar analysis comparing smart suits and sensing electrode stickers to wrist/ankle bands was also conducted (Methods and Results are reported in S6).”, in the methods section’s analysis section under Sticker sensing electrodes versus smart suits, so that it is more of a sub/complementary analysis.

**Reviewer #3**

**I am overall satisfied with the revisions and I thank the authors for their changes. I have only a few minor remaining comments:

Comment 1: Without revealing anything about this study I contacted a British English speaking colleague as well as a non-native English-speaking colleague about what "video" means to them in this context. While the BrEng colleague's judgment fell well in line with the authors' the non-native English speaking colleague's judgments fell in line with my own: that "video" without further specification is ambiguous as to an image-only stream or an audio-visual stream. Please add just a very short clarification to avoid dialectical issues in interpretation.**
Response: We have added “image-only video” where appropriate throughout the paper:

- Aims:
- Privacy section of methods:
- Results section: “Static versus body **image-only video** recording devices”.
- Results Privacy section overview paragraph: “In-home remote infant monitoring studies, particularly those with highly identifiable **image-only video** and audio recording, raise privacy issues.”
- Discussion: “Static versus body **image-only video** recording devices” section title and we added to the first line: “Caregivers indicated similar levels of usage likelihood for static cot cameras versus on-body camera **for image-only video** recording”
- Discussion: **“Image-only** video versus audio recording with/without privacy preservation” section title. “And Similar to smartphones, audio or **image-only** video recording not only contribute contextualising data, but additional informative data”…. “The lack of preference to either image-only video or audio”

**Comment 2: There is still at least one case of "stakeholder" that needs to be replaced: "it is critical to gather stakeholder’s views" >> should be "caregivers'" or perhaps "participating families'"; please check again for other cases.**
Response: Corrected to caregivers

**Comment 3: The context for sending the weblink comes after it's first mentioned (i.e., after information about participant responses), which may be confusing. Could the recruitment information come first?**
Response: We have moved the Recruitment procedure to come before Participants section.

**Comment 4: Please mention "UK" again at the top of the summary as it's appropriate to specify scope in a discussion section**
Response: Added “in the UK” to Fig 9. Description. And “ in-home monitoring with infant participants in the UK” to the figure title.

**Comment 5: There are a number of minor typos/grammatical errors throughout the manuscript (e.g., some possessive apostrophes, extra capitalization, extra/missing spaces).**
Response: With careful review, we have now corrected all those we are aware of.

**Reviewer #4**

**Comment 1: INTRODUCTION: Minor comment, Line 122: This sentence is slightly confusing as written. Adding a comma after "lab setting" would make it more readable. "In a lab setting environments are controlled, but in a home setting the context in which the data is being collected can vary dramatically both between infants (e.g. single child, multi-generational household, etc) and within infant across a day (e.g. dinner time, playtime, bedtime etc)."**
Response: The comma has been added.

**Comment 2: Minor comment, Line 144: Please revise this sentence for clarity: " A previous parent opinion survey established privacy-preserving techniques (e.g. the implementation of computer algorithms to automatically extract behaviour markers independent of identity), to only minorly improve willingness to participate in the collection of identifiable measures."**
Response**:** We have reviewed this sentence and hope it is now clear. “A recent report on a USA-based sample of parents indicated privacy-preserving techniques to minorly improve willingness to participate in the collection of identifiable data on their infant (29). Such privacy-preserving techniques included the implementation of computer algorithms to automatically extract measures of behaviour and remove identifiable information (29).” **Comment 3: METHODS: Minor comment, Line 222: Some people may oppose the demographic results being in the methods section as opposed to the results section. Please make sure this aligns with the journal guidelines**
Response: We have divided the “Participants” section. In the methods we explain how many people participated and refer to the attrition SM. The demographics part of that paragraph has now been moved to the beginning of the results section under “Sample Demographics”

**Comment 4: Minor comment: Is the abbreviation B.A.M.E necessary in Table 1? Can this be spelled out?**

Response: We have changed this to “Black, Asian and Minority Ethnic Group”.

**Comment 5: Minor comment, Line 278: Please add a reference for researchers who may want replicate the text mining analysis. In the supplement there are reference citations, but I do not see actual references.**
Response: Reference has been added (Smith A. Leximancer Pty Ltd [Internet]. Brisbane, Australia; 2009. Available from: https://info.leximancer.com) **Comment 6: RESULTS: Minor comment: Some sentences start with numerical percentages. Please make sure this aligns with journal guidelines.**
Response: We have now corrected this throughout the paper.  **Comment 7: DISCUSSION: Major comment: Given the limitations of this text mining analysis, it would be nice to put the results about caregivers' concerns for safety in the context of the existing literature. Is there literature finding similar or different results regarding safety?**
Response: This has been noted in previous literature, we have added (line 868) “This finding was consistent with a previous qualitative report on potential barriers for participation with ambulatory infant sensing devices; caregivers expressed concerns about the comfort of the physical placement of the sensor on their child (29). Though such concerns are likely to be addressed by manufacturers of the technologies as well as local ethics boards prior to implementation, future researchers should explicitly state the safety of these measures during advertising/consent to improve participant uptake.”

**Comment 8: Minor comment, Line 830: Canvass is misspelled: "In order to comprehensively cavass caregivers’ opinions on remote monitoring technologies and study designs, we asked a large number of question."**

Response: Corrected.

Kind Regards,

Laurel Fish and Prof. Emily Jones
